# Isolation and Identification of Phytocompounds from *Maytenus dhofarensis* and Their Biological Potentials

**DOI:** 10.3390/molecules28166077

**Published:** 2023-08-15

**Authors:** Fatma Al-Rubaiai, Zakiya Zahran Al-Shariqi, Khalsa S. Al-Shabibi, John Husband, Asmaa M. Al-Hattali, Marcia Goettert, Stefan Laufer, Younis Baqi, Syed Imran Hassan, Majekodunmi O. Fatope

**Affiliations:** 1 Department of Chemistry, College of Science, Sultan Qaboos University, Al Khod, P.O. Box 36, Muscat 123, Oman; s87268@student.squ.edu.om (F.A.-R.); johnh@squ.edu.om (J.H.); baqi@squ.edu.om (Y.B.); 2 Department of Pharmaceutical and Medicinal Chemistry, Institute of Pharmacy, Eberhard Karls Universität Tübingen, D-72076 Tübingen, Germanystefan.laufer@uni-tuebingen.de (S.L.); 3 Tübingen Center for Academic Drug Discovery (TüCAD2), D-72076 Tübingen, Germany

**Keywords:** *Maytenus dhofarensis*, secondary metabolites, biological activities

## Abstract

*Maytenus dhofarensis* Sebsebe (Celestraceae) is a naturally growing shrub in Oman. It is not a reputed medicinal plant in Oman, but it is regionally endemic and causes shivering attacks on goats that graze on it. The chemical investigation of the hexane and chloroform extracts of the fruits and stems of *M. dhofarensis* afforded dihydro-β-agarofuran-type sesquiterpene pyridine alkaloid (**1**), lupanyl myristoate (**2**) and lignanolactone (**3**). Compounds (**1**–**3**) are new isolates from *M. dhofarensis*. The structures of these compounds were assigned through comprehensive IR, NMR, and ESI-MS analyses, and the relative configurations of compounds **1** and **3** were deduced from density function theory (DFT) calculations and NMR experiments. Compound **1** was assayed against the kinase enzyme and showed no inhibition activity for p38 alpha and delta at a 10 µM test concentration. Compound **3** inhibited the 2,2′-diphenyl-1-picrylhydrazyl radical (DPPH) by 69.5%, compared to 70.9% and 78.0% for gallic acid and butylated hydroxyanisole, respectively, which were used as positive controls.

## 1. Introduction

Plants of the genus *Maytenus* (Celestraceae) are widely distributed in tropical and subtropical regions of the world and about 800–1300 species [1] are known. Several members of this genus are used in traditional medicine to treat cancer [2], gastric ulcers [3], and arthritis [4]. *Maytenus* species are known to contain a diverse group of triterpenoids [5,6,7], flavonoids [8,9], tannins [10], lignans [11,12], dihydro-β-agarofurans [4,13,14] and sesquiterpene pyridine alkaloids [15,16,17] that display remarkable structural diversities and cytotoxicity [18,19,20,21,22], as well as insecticidal [23], antitumor-promoting [13], MDR-reverting [24,25,26], antitubercular [27], neuroprotective [28], immunosuppressive [29], anti-HIV [30], anti-inflammatory [18], and medicinal properties [3,31,32,33,34,35,36,37].

**Figure 1:**
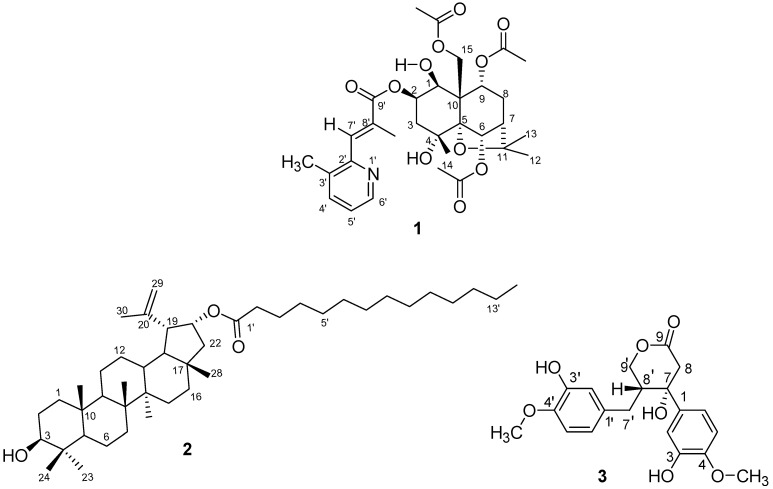
Structures of isolated compounds **1–3.**

*Maytenus dhofarensis* is a regionally endemic spiny shrub found growing naturally in the Dhofar region of Oman [38]. It is not a reputed medicinal plant in Oman but causes shivering attacks on goats that graze on it. To date, no reports on its secondary metabolites have previously been described. The lack of elaborate phytochemical and pharmacological work on this species stimulated our interest in examining the plant for structurally novel compounds and bioactivity. Herein, we report the isolation and structural characterization of a new dihydro-β-agarofuran-type sesquiterpene pyridine alkaloid (**1**), lupanyl myristoate (**2**), and lignanolactone (**3**) (Figure 1) from the fruits and stems of the plant, and the bioassay results of the more-abundant isolates for their antioxidant and kinase-inhibitory activities. Their structures were elucidated from the interpretation of spectral data and the relative configurations of compounds **1** and **3** were determined from observed and calculated chemical shift values for their diastereoisomers using DP4+ probability [39,40] analysis. Compound **3** showed 2,2′-diphenyl-1-picrylhydrazyl radical (DPPH)-scavenging activities. 

## 2. Results and Discussion

Compound **1** was isolated as a colorless gum, and it produced an alkaloid-positive test with Dragenddorrff’s reagent. Its molecular formula (C_31_H_41_NO_11_) was deduced from the ESIMS ion peak at *m/z* 645.1000 [M + CH_3_CN + H]^+^ (calc. *m/z* 645.1000 for C_33_H_45_N_2_O_11_), corresponding to an acetonitrile-adduct ion cluster with one proton. The UV spectrum exhibited characteristic absorption bands for aromatic moiety (λ_max_ at 283 (1.6), 275 (1.9), and 246 (3.0) nm). The infrared (IR) spectrum displayed absorption bands at 3280 (broad), 1745, 1708, and 1596 cm^−1^ for free hydroxyl, multi-carbonyl esters, and an aromatic ring, respectively. Its ^1^H NMR spectrum data (Table 1) revealed three acetyl methyl groups at δ_H_ 1.78 (s), 2.08 (s), and 2.28 (s); four oxygenated methines at δ_H_ 5.53 (H-2, m), 6.22 (H-6,s), 5.28 (H-9, d, *J* = 6.8 Hz) and 5.44 (H-1, d, *J* = 3.2 Hz); a hydroxyl proton at δ_H_ 3.10; and one set of oxygenated methylene signals at δ_H_ 4.92 (H-15a, d, *J* = 12.8 Hz) and 4.39 (H-15b, d, *J* = 13.6 Hz), respectively. The signal at δ_H_ 2.35 was assigned to the aliphatic methine proton (H-7). There were also resonances for four sets of aliphatic methylene protons at δ_H_ 2.02 (H-3a, dd, *J* = 3.2, 15.1 Hz), 2.18 (H-3b, dd, 5.3, 16.0 Hz), 2.19 (H-8a, m) and 2.60 (H-8b, ddd, *J* = 3.8, 11.1 15.9 Hz) and five tertiary methyl protons at 1.52 (H-13, s), 1.57 (H-12, s), 1.49 (H-14, s), 1.81 (H-10′, s), and 1.85 (H-11′, s). The vinylic signal at δ_H_ 6.93 corresponded to the olefinic proton H-7′, which appeared as a doublet of a quartet (*J* = 7.0, 1.3 Hz). The ^1^H NMR spectrum data revealed the presence of three 2,3 disubstituted pyridine protons at δ_H_ 7.48 (H-5′, dd, *J* = 8.1, 7.6 Hz), 7.58 (H-4′, dd, *J* = 7.4, 2.5 Hz) and 8.18 (H-6′, dd, *J* = 8.3, 1.2 Hz). Additional signals for four ester carbonyls at δ_C_ 166.5, 169.8, 169.5, and 170.7; three oxygenated quaternary carbons at δ_C_ 69.9 (C-4), 84.7 (C-11), and 91.1 (C-5); one olefinic quaternary carbon at δ_C_ 129.8 (C-8′); and two aromatic quaternary carbons at δ_C_ 127 (C-3′) and 166.1 (C-2′) were observed in the ^13^C NMR spectrum (Table 1). Taken together, these data indicate that compound **1** is a dihydro-β-agarofuran-type sesquiterpene pyridine alkaloid [16,18]. A polyoxygenated dihydroagarofuran skeleton was determined by the ^1^H–^1^H COSY cross-signals for H-1/H-2, H-2/H-3, H-7/H-8, and H-8/H-9, coupling systems and the following HMBC interactions: H-1/ C-2, C-9, C-10, C-15, and C-9′; H-9/ C-5, C-7, C-8 C-10 and C-15; H-15/ C-1, C-5 and C-9; and H-6/ C-5, C-7, C-8, C-10 and C-11. The free hydroxyl groups were located at C-1 (δ_C_ 70.6) and C-4 (δ_C_ 69.9) by a comparison of the observed ^13^C NMR chemical shift with reported values [16,18] and the HMBC cross-signals of OH (δ_H_ 3.1) with C-4, C-5, and C-14, respectively.

The 2,3-disubstituted pyridine core unit was confirmed by ^1^H-^1^H COSY correlations and HMBC interactions. In the HMBC interactions (Table 1), H-5′ (δ_H_ 7.48) showed interaction with a methine carbon signal at δ_C_ 127.5 (C-3′). Signals at δ_H_ 8.18 (H-6′) and 6.93 (H-7′) showed interactions with δ_C_ 166.5 (C-2′). Also, the signal at δ_H_ 7.58 (H-4′) showed interaction with δ_C_ 130.19 (C-6′). The ^1^H−^1^H COSY spectrum of compound **1** revealed a separated spin−spin system (H-4′/H-5′/H-6′).

The relative configuration of **1** was established by the 2D NOESY spectrum and by comparing it with previous studies [16,18]. DFT-predicted chemical shifts for 9*R* and 9*S* diastereomers were compared with the observed values for the isolated compound, and the best match using the DP4+ probability [39] favored the 9*R* configuration for compound **1**. The NOESY spectrum also did not show any correlation between H-1 and H-9, supporting the *9R* configuration assignment. Predicted chemical shift values are listed in Table S1, while the results of the DP4+ analyses are summarized in Table S2. Based on the above assignments, the structure of compound **1** was identified as a dihydro-β-agarofuran-type sesquiterpene pyridine alkaloid, which was named *Maytendhofarene* (Figure 1).

**Figure 2:**
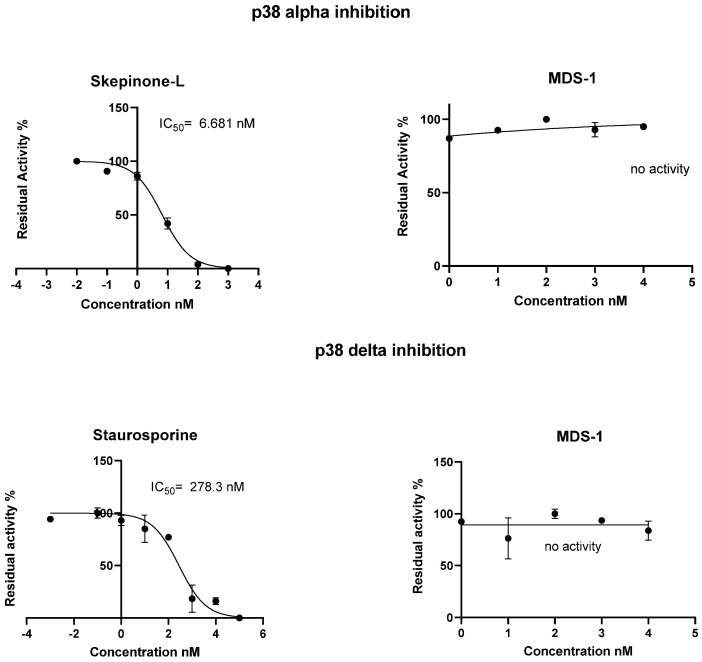
Kinase Inhibition of Compound **1** against p38 alpha and delta.

Compound **1** was investigated for kinase-inhibition activity using the homogenous time-resolved fluorescence (HTRF) detection kit [41] but showed no inhibition activity for p38 alpha and delta at a 10 µM test concentration (Figure 2). p38 MAP kinases have been implicated in a wide range of complex biological processes, such as cell differentiation and proliferation, cell death, cell migration, and invasion [42]. The dysregulation of p38 MAPK is associated with diverse diseases such as chronic inflammation and cancer and can act as a tumor suppressor or tumor inducer [43].

Compound **2,** whose molecular formula was identified as C_44_H_76_O_3_ from the HRESIMS sodium-adduct ion cluster at 675.6628 [M + Na]^+^ (calcd. for C_44_H_76_O_3_Na 675.5692), was obtained as a white solid (m.p. = 66-68 °C). The FTIR spectrum showed absorption bands at 3373 (broad), 2916, 2849, and 1702 cm^−1^ characteristic of hydroxyl, alkene, and ester groups, respectively. The ^1^H NMR spectrum (Table 2) displayed six tertiary methyl groups (each, 3 H, s) at δ_H_ 1.03, 0.94, 0.87, 0.85, 0.84, 0.78 and one isopropenyl methyl at δ_H_ 1.68 (3H, s). Two exocyclic vinyl protons resonated at δ_H_ 4.69 (1H, s) and 4.57 (1H, s). Two oxymethine signals at δ_H_ 4.53 (1H, dd, *J* = 14.0 and 7.0 Hz) and δ 3.99 (1H, m) and a typical lupene H_β_-19 resonance at δ_H_ 2.38 (1H, dd, *J* = 14.0 and 7.0 Hz) were also observed. These signals indicated a dihydroxy lupene triterpene substructure with one hydroxyl group masked as an ester for compound **1**. The multiplicity and ^1^H-^1^H COSY connectivity of the H_β_-19 signal at δ_H_ 2.38 (dd, *J* = 14.0 and 7.0 Hz) to δ_H_ 3.99 (H-21) and δ_H_ 1.35 (H-18) showed that C-19 is flanked by oxymethine and methine groups. The resonances at δ_H_ at 1.42 and 1.32 were assigned to H-22a and H-22b based on their connectivity to δ_H_ 3.99 (H-21) in the ^1^H-^1^H COSY map. The exocyclic alkene group was confirmed by the resonances at δ_C_ 151.1 (C-20) and 109.5 (C-29) in the ^13^C NMR DEPT spectrum. The assignment of the resonances of the lupene substructure (Table 2) allowed the signals of the fatty ester to be easily recognized and assigned. The positions of the hydroxy and myristoxy groups were confirmed by H-3/H-2 correlation in the ^1^H-^1^H COSY, and H-3/C-2, H-3/C-4, H-3/C-23, H-3/C-24, H-21/C-1′, H-21/C-19, H-21/C-22 in the HMBC. Additional HMBC interactions between δ_H_ 2.38 (H-19) and δ_C_ 38.2 (C-13), 48.1 (C-18), 151.1 (C-20), 68.4 (C-21), 35.7 (C-22), 109.5 (C-29), 18.1 (C-30), and 173.0 (C-1′) corroborated the assigned structure. The orientations of C-3 hydroxyl and C-21 myristoxy groups were determined as β and α based on coupling constants or chemical shift values [44] of H-3 and H-21. Compound **2** is a 3β, 21α-dihydroxylup-20(29)-ene, with the C-21 hydroxyl group masked as an ester of myristic acid and named 3β-hydroxy-21α-myristoxylup-20(29)-ene (**2**). 

Compound **3** was isolated from the CHCl_3_ extract of *M. dhofarensis*. It was a solid (m.p. = 75–80 °C) and exhibited IR absorption bands for an OH stretch (3982 and 3397 cm^−1^, broad), ester (1738 cm^−1^), and benzene ring (1599, 1508 cm^−1^). The molecular formula was determined to be C_20_H_22_O_7_ from the HRESIMS sodium-adduct ion cluster at *m/z* 397 [M + Na]^+^ and a protonated dimer molecule ion at *m/z* 749.2809 [2M + H]^+^ (calcd. for C_40_H_45_O_14_ 749.2812). This formula is consistent with the presence of ten degrees of unsaturation. The ^1^H NMR spectrum (Table 3) showed signals for six aromatic protons and some phenolic hydroxy, two methoxy, one methane, and three methylene groups for compound **3.** It also displayed isochronous signals at δ_H_ 4.04 and 3.99, δ_H_ 3.10, and 2.91, and δ_H_ 2.59 and 2.49 for oxymethylene, acetoxymethylene, and benzylmethylene protons (Table 3). All the methylene protons in compound **3** are thus diastereotopic atoms. A methine proton at δ_H_ 2.52 (H-8′) showed spin–spin couplings to the oxymethylene protons (H-9′a and H-9′b) and benzylmethylene protons (H-7′a and H-7′b). The diastereotopic acetoxy protons were only coupled to each other in the ^1^H-^1^H COSY map and must be flanked by quaternary carbons. The interpretation of the IR, ^1^H NMR chemical shifts, and coupling patterns suggested a β-hydroxy-β-phenyl-γ-benzyl δ-valerolactone substructure for compound **3**. The two methoxy groups resonated as singlets at δ_H_ 3.64 and 3.85, and the six aromatic protons constituted two separate ABX coupling systems with characteristic splitting patterns of aromatic protons in 1,3,4 relative positions. Each of the four aromatic protons resonated as a doublet (*J* = 7.9 Hz) at δ_H_ 6.84, 6.82, 6.63, and 6.62, and the other aromatic protons as broad singlets at δ_H_ 6.69 and 6.60, respectively. The aromatic signals and spin–spin couplings are consistent with the presence of two 3, 4-di-substituted phenyl units in compound **3**. The ^13^C NMR Broad Band and DEPT experiments resolved twenty carbon resonances for compound **3,** which included one carbonyl at δ_C_ 179.1 (C-9) and two tri-substituted aromatic units (Table 3). The spectra also displayed the following carbon signals: one oxymethylene at δ_C_ 70.7 (C-9′), one acetoxy methylene at δ_C_ 42.4 (C-8), one benzylmethylene at δ_C_ 31.9 (C-7′), one methine at δ_C_ 44.2 (C-8′), one oxygenated quaternary carbon at δ_C_ 76.9 (C-7), and signals for two methoxy groups at δ_C_ 56.4 and 56.3, respectively. The assignment of methoxy groups to C-4 and C-4′ was complemented by connectivity between δ_H_ 3.85 and δ_C_ 144.7 (C-3) and δ_C_ 147.0 (C-4) or δ_H_ 3.64 and 147.0 (C-3′) and 145.4 (C-4′) in HMBC. All the methylene and methine protons showed connectivity to the quaternary carbon resonance at δ_C_ 76.9 (C-3) in the HMBC. Additional HMBC connectivities between the protons of benzylmethylene [δ_H_ 2.59 (H-7′a) and 2.49 (H-7′b)] and δ_C_ 126.5 (C-1′), 121.9 (C-6′), and 111.9 (C-2′); oxymethylene [δ_H_ 4.04 (H-9′b) and 3.99 (H-9′a)] and δ_C_ 179.1 (C-9); and acetoxymethylene [δ_H_ 3.10 (H-8a) and 2.91 (H-8b)] and δ_C_ 179.1 (C-9), 44.2 (C-8′), and 130.7 (C-1) resonances established a lignanolactone structure for compound **3**. Positions C-7 and C-8′ are chirality centers. The peripheral diamagnetic anisotropy of the phenyl groups at C-7 and C-7′ should cause H-8′ and H-7′, in particular, to resonate at a lower field when the configuration of H-8′ is *R* (Figure S23), and the phenyl groups at C-7′ and C-7 are quasi syn-periplanar to H-8′, as revealed by the molecular model of compound **3**. DFT-predicted chemical shifts for H-7*R*, H-8′*R* and H-7*S*, and H-8′*R* diastereomers were compared (Table S3) with the observed values for the isolated compound, and the best match (98.9%) using the DP4 probability [40] favored H-7*S*, H-8′*R* configuration for compound **3**. Compound **3** demonstrated radical scavenging property, inhibiting [45] DPPH by 69.5%, compared to 70.9% and 78.0% for gallic acid and butylated hydroxyanisole, which were used as controls.

## 3. Experimental Section

### 3.1. General Experimental Procedures

IR spectra were obtained with a Nicolet FT-IR spectrometer. ^1^H and ^13^C NMR spectra were recorded in CDCl_3_ with Bruker Advance NMR spectrometer operating at 700 MH with TMS as the internal standard. ESIMS was recorded on a Quattro Ultima Platinum Tandem quadrupole mass spectrometer (Micromass, Wilmslow, UK). ESIMS data were acquired on an Agilent 6400 Triple Quad LC/MS and using HRESIMS. The column chromatography (CC) was performed using EM Science Silica gel 60 (70–230 mesh ASTM). Whatman precoated silica-gel (60A K6F) analytical plates (20 × 20 cm) were used for TLC, with compounds visualized by a UV lamp and spraying with 10% (*v/v*) H_2_SO_4_ or Molybdophosphoric acid-isopropanol followed by heating. All absorbance measurements were recorded using a Shimadzu UV spectrophotometer.

#### Solvents and Reagents

The solvents used in this investigation were of analytical grade. The hexane, diethyl ether, ethyl acetate, and deuterated chloroform (CDCl_3_) were purchased from Sigma Aldrich (Dorset, UK). Chloroform and ethanol were purchased from BDH (Hampshire, UK). Moreover, 2,2′-diphenyl-1-picrylhydrazyl radical (DPPH), butylhydroxy anisole, and gallic acid were purchased from Sigma Aldrich (Steinheim, Germany).

### 3.2. Plant Material

*M. dhofarensis* was collected from Gogeb in Salalah in the Dhofar region, Oman (GPS coordinates: 17°12′42″ N, 54°6′29″ E) in December 2018 at an altitude of 800 m. The plant was identified by Dr. S.A. Ghazanfar at the Royal Botanic Gardens, Kew Richmond, UK, and Dr. Amina A. Al Farsi at the Department of Biology, College of Science, Sultan Qaboos University, Muscat, Oman. A voucher specimen (SQUH00006216) is kept in the Herbarium of the Department of Biology, College of Science, Sultan Qaboos University, Muscat, Oman. 

### 3.3. Extraction and Isolation 

The fruits of *M. dhofarensis* were dried in a hot room (at 42 °C) for 3 weeks in the Department of Chemistry, Sultan Qaboos University, and the seeds were separated from the calyx and milled to give 576 g of powdered seed. The seeds (288 g) were extracted with chloroform (2 × 1800 mL) by maceration at room temperature for three days each and concentrated under vacuum at 25–30 °C to give a gummy residue (208.6 g). The column chromatography of a portion of the chloroform extract (44.75 g) on silica gel (895 g), using gradient mixtures of *n*-hexane—CHCl_3_, CHCl_3,_ and CHCl_3_—EtOH as eluent, gave a variable number of fractions, which were combined based on their TLC profiles. 

Preliminary cytotoxicity testing using the brine shrimp test [46] (BST) revealed that (*Maytenus dhofarensis*) *Md*-seed-CHCl_3_-F73 is the most active fraction. This fraction (*Md*- seed-CHCl_3_-F73) was chromatographed on a silica gel (91 g) column (2.5 cm × 35 cm), starting with *n*-hexane, and then the polarity was gradually increased using ethyl acetate. Compound **1** (23.4 mg; eluent hexane-diethyl ether 3:1) was obtained as a pure compound from subfraction F73-82 (384 mg) and purified by multi-column chromatography using hexane-diethyl ether mobile phase.

A portion of the dried and powdered calyx (275 g) of *M. dhofarensis* was extracted with hexane by Soxhlet (2 × 600 mL) for four hours. The solvent was removed in a vacuum to yield a hexane extract (11.5 g). The separation of the extract was undertaken using column chromatography with silica gel (120 g) and using hexane and gradient mixtures of hexane-ethyl acetate with a collection of 100 mL fractions. TLC analysis using hexane: EtOAc (1:4) as the eluting system allowed fractions (*M.d*-calyx-H-40 to *M.d*-calyx-H-50) to be combined with *M.d*-calyx-Hexane-50 (441 mg) and purified on silica gel (105 g). Then, they were eluted using gradients of diethyl ether (DEE) in hexane as a mobile phase to produce fractions 1–33. Fraction *M.d*-calyx-Hexane-50-9 (128.7 mg) eluted with hexane: DEE (1:1.5) was further purified on silica gel (54 g), eluting with gradients of DEE in hexane to afford *M.d*-calyx-Hexane-50-9-1 to *M.d*-calyx-Hexane-50-9-156. Fractions *M.d*-calyx-Hexane-50-9-91 to *M.d*-calyx-Hexane-50-9-93 gave (5.0 mg; eluent hexane-DEE 2:1) pure compound **1**.

The dried and powdered twigs *M. d* (2.0 kg) were extracted with hexane (5 L) for one week to give a residue (12.7 g) after solvent removal. The defatted plant material was re-extracted with CHCl_3_ (5L× 2) for two weeks and then concentrated under vacuum to yield a brown residue (59.5 g). 

The hexane extract (12.0 g) was chromatographed on a silica-gel (150 g) CC column (3.2 cm × 46.5 cm) eluted with hexane in a gradient mode with CHCl_3_ to give fractions MDp-1 to MDp-107. Fractions were combined in groups based on their TLC similarity. Fractions MDp-44 and MDp-45 were combined with MDp-45 (1.7 g) and loaded on a silica-gel (100 g) column (2.5 cm × 30 cm) (eluted with a Hex/ EtOAc gradient, yielding 58 fractions (MDp-45-1 to MDp-45-58). Fractions MDp-45-10 to MDp-45-12 (eluent hexane-EtOAc 10:2) were combined with MDp-45-12 (0.34 g) due to their TLC similarity. Fraction MDp-45-12 was further purified by CC with Hexane-EtOAc in increasing order of polarity on Si to produce a pure compound **2** (2.5 mg; eluent Hexane-EtOAc (10:1)**.**

The CHCl_3_ extract of the powdered twigs (25.0 g) was fractionated with silica-gel (180 g) CC using hexane, CHCl_3_, EtOAc, and EtOH as eluent to give a primary fractioning of 36 fractions (M.d-1 to M.d-36). The purification of M.d-19 (2.3 g, eluent hexane 100%) with the gradient of EtOAc in hexane gave M.d-19-1 to M.d-19-79. M.d-19-36, eluted by hexane: EtOAc (7:3), showed a single spot on TLC and gave a pure compound **3** (2.3 mg).

***Maytendhofarene* (1):** colorless gum. IR υ_max_ cm^−1^ 3280, 2921, 1745, 1708, 1594, 1371, 1224, 1067; UV (CHCl_3_) λ_max_ 283 (1.6), 275 (1.9) and 246 (3.0); ^1^H NMR (CDCl_3,_ 700 MHz); and ^13^C NMR (CDCl_3,_ 176 MHz) data are given in Table 1; ESIMS: *m/z* 645.1000 [M + CH_3_CN + H]^+^ (calc. 645.3000 for C_33_H_45_N_2_O_11_). 

**3β-Hydroxy-21α-myristoxylup-20(29)-ene** (**2**): white solid. mp 66-68 °C; FTIR υ_max_ 3373 (br), 2916, 2849 and 1702 cm^−1^ ;^1^H (CDCl_3,_ 700 MHz); and ^13^C NMR (CDCl_3,_ 176 MHz) data are given in Table 2; HRESIMS *m/z* 675.6628 [M + Na]^+^ (calcd for C_44_H_76_O_3_Na 675.5692). 

**4,4′-Dimethoxy-3,3′,7-trihydroxy-7,8′-lignano-9,9′-lactone** (**3**)**:** whitish-yellow solid. mp 75-80 °C; FTIR υ_max_ (cm^−1^) 3982, 3397 (hydroxyl), 1738 (ester) and 1599 and 1508 (aromatic ring); ^1^H NMR (CDCl_3,_ 400 MHz); and ^13^C NMR (CDCl_3,_ 100 MHz) data are given in Table 3; HRESIMS *m/z* 397 [M + Na]^+^, and *m/z* 749.2809 [2M + H]^+^ (calcd for C_40_H_45_O_14_ 749.2812).

### 3.4. Computational Studies of Compounds ***1*** and ***3***

Density functional theory (DFT) was used to predict the NMR chemical shifts for the diastereomers of compound **1** (9*R* and 9*S*) and compound **3**, (7*R*-8′*R* and 7*S*-8′*R*). For each, conformers up to an upper limit of 10 kcal/mol were identified using the MMFF force field as implemented by MarvinView software (version 17.2.6.0) [47]. The selected rotamers were optimized using Gaussian (G09W) software (Gaussian 09, revision E.01) [40] at the B3LYP/6-31G+(d,p) (gas-phase) level of theory, and subsequent frequency calculations confirmed the absence of any imaginary frequencies in the minimized structures. Isotropic shielding constants were calculated at the mPW1PW91/6-311+G(d,p)/PCM level using the GIAO method. The predicted values for each diastereomer were averaged using a Boltzmann weighting, and for each compound, the unscaled shielding constants were compared in the Bayesian-based DP4+ analyses utilizing the Excel file provided by the Sarotti group [39].

### 3.5. Biological Assays 

#### 3.5.1. Homogenous Time-Resolved Fluorescence (HTRF) Kinase Assay

Compound **1** was diluted with a kinase buffer (with the p38 alpha enzyme or p38 delta enzyme) arising out of a stock solution of 10 mM in MDSO, giving a final concentration of 10 µM. Subsequently, 10 µL of the compound dilution was added to the enzyme, producing a final volume of 20 µL. Instead of a compound, 10 µL of KB was distributed in the wells of negative (NSB) and positive controls (STIM). The 96-well non-binding plate was centrifuged before a preincubation for 10 min at 37 °C, gently shaking at 150 rounds. After the preincubation, an ATP/ATF 2 solution was added to the wells and incubated again for 30 min at 37 °C. The assays were performed using the homogenous time-resolved fluorescence (HTRF) detection kit (Cisbio, Bedford, MA) by adding 10 µL of the HTRF detection solution (2.5 µL of PAb Anti-phospho ATF 2-Eu cryptate (1:400); 5 µL of MAb Anti GST-d2 (1:200); and 992.5 µL of HTRF detection buffer). The plate was incubated for 30 min at room temperature in the dark. The HTRF signal was read from the Victor Nivo^®^ and calculated as the ratio of signal from the 665 nm (acceptor) and 615 nm (donor) channels and multiplied by 10,000. The percent activity was calculated by normalizing the HTRF signal from each sample well to the mean HTRF signal from the DMSO-only control wells, using the following equation:

Inhibition (%) =100 − ((ODsample − NSB)/(ODstim − NSB)) × 100%

#### 3.5.2. Antioxidant Assay Activity Using 2,2′-Diphenyl-1-picrylhydrazyl (DPPH) Radical-Scavenging Method

The free-radical scavenging activity of compound **3** was determined using the protocol reported by Morelli [45] with slight modifications: 8.65 mg of compound **3** was dissolved in CHCl_3_ (1 mL), and an aliquot of this was diluted with CHCl_3_ to give a solution of 0.4 mg/2.0 mL of compound **3**. This solution was mixed with 2.0 mL of 100 µM DPPH solution prepared by dissolving 2 mg DPPH in 50 mL of 25% aqueous ethanol. Butyl hydroxyl anisole (BHA) and gallic acid solutions were used as positive controls, while 2.0 mL of 25% aqueous ethanol solution of 100 µM DPPH mixed with 2 mL of CHCl3 served as the blank solution. The absorbance at 517 nm of all prepared solutions was determined after 15 min of incubation in the dark against the blank solution. 

The ability to scavenge DPPH was expressed as a percentage inhibition (% IP) of the DPPH radical.

Inhibition (%) = [(Absorbance of control – Absorbance of sample)/Absorbance of control] × 100%

## 4. Conclusions 

Previously uninvestigated endemic *M. dhofarensis* yielded three new compounds (**1**-**3)**, which are similar to the compounds found in the genus *Mayteneus.* However, compound **1** is structurally unique. It is a dihydro-β-agarofuran-type sesquiterpene pyridine alkaloid that differs structurally from the vast array of Celastraceous macrocyclic dihydro-β-agarofuran sesquiterpene pyridine alkaloids [48] due to the absence of a pyridine dicarboxylic acid macrocyclic bridge. This is structurally significant for an endemic plant of the genus *Mayteneus*. Compound **1** lacked kinase-inhibitory activity as hoped for in the original design of this work. It was obtained in the fruit and also detected in the alcohol extracts of the stem. The fruit is toxic to goats, and whenever a goat grazed the fruits, it fell ill with shivering attacks. Compound **3** is a new addition to the secondary metabolites from this genus. It was isolated from the stem and showed radical scavenging activity at a level comparable to gallic acid. This might help the plant to overcome oxidative stress.

## Figures and Tables

**Table 1:** ^1^H NMR (700 MHz) and ^13^C NMR (176 MHz) Data for Compound **1** in CDCl_3._

Position	δ_C_ (ppm)	δ_H_ (ppm) (*J* in Hz)	HMBC (^1^H → ^13^C)
1	70.6	5.44, d (3.2)	2, 9′, 10, 15
2	68.3	5.53, m	-
3	42.3	H_a_, 2.02, dd (3.2, 15.1) H_b_, 2.18, dd (5.3, 16.0)	-
4	69.9	-	-
5	91.1	-	-
6	78.7	6.22, s	5, 7, 8, 10, 11, 16
7	49.2	2.35, m	-
8	34.6	H_a_, 2.19, m H_b_, 2.60, ddd (3.8, 11.1, 15.9)	-
9	68.2	5.28, d (7.2)	5, 7, 8, 10, 15
10	54.9	-	-
11	84.7	-	-
12	29.5	1.57, s	7, 11, 13
13	25.7	1.52, s	7, 11
14	24.9	1.49, s	3, 5
15	65.5	H_a_, 4.92, d (12.8) H_b_, 4.39, d (13.6)	5, 9 5, 10
2`	166.1	-	-
3`	127.5	-	-
4`	133.4	7.58, dd (7.4, 2.5)	1′
5`	128.7	7.48, dd (8.1, 7.6)	3′
6`	130.2	8.18, dd (8.3, 1.2)	2′, 4′, 6′
7`	140.2	6.93, dq (7.0, 1.3)	2′, 10′, 11′
8`	129.8	-	-
9`	170.7	-	-
10`	11.9	1.81, s	-
11`	14.7	1.85, s	9`
OAc-6	*CH_3_:* 20.6	1.78, s	-
	*C=O:*169.5	-	
OAc-9	*CH_3_:* 21.4	2.28, s	-
	*C=O:*166.5	-	-
OAc-15	*CH_3_:* 21.2	2.08, s	-
	*C=O:*169.8	-	-
OH	-	3.10	5, 7, 14

**Table 2:** ^1^H NMR (700 MHz) and ^13^C NMR (176 MHz) Data for Compound **2** in CDCl_3._

Position	δ_C_ (ppm)	δ_H_ (ppm) (*J* in Hz)	HMBC (^1^H → ^13^C)
1a	38.5, CH_2_	0.98 ^a^	
1b		1.61 ^a^	
2	23.9, CH_2_	1.62 ^a^, m	
3	81.6, CH	4.53, dd (14.0, 7.0)	2, 4, 23, 24
4	37.9, C		
5	55.6, CH	0.85 ^a^	
6	21.1, CH_2_	1.36 ^a^	
7	41.8, CH_2_	2.41, 2.39, dd (14.0, 7.0)	
8	43.1, C		
9	50.5, CH	1.29 ^a^, m	
10	37.2, C		
11	25.6, CH_2_	1.32	
12	27.6, CH_2_	0.88, t (7)	
13	38.2, CH	1.61 ^a^, m	
14	40.9, C		
15	34.3, CH_2_	1.35 ^a^, m	
16	40.1, CH_2_	1.15–1.30 ^a^, m	
17	42.9, C		
18	48.1, CH	1.35 ^a^, m	
19	48.41, CH	2.38 dd (14.0, 7.0)	1′, 13, 18, 20, 21, 22, 29, 30
20	151.1, C		
21	68.4, CH	3.99, m	
22a	35.7, CH_2_	1.42 ^a^, m	
22b		1.32 ^a^, m	
23	28.2, CH_3_	1.03, s	
24	18.3, CH_3_	0.78, s	
25	16.1, CH_3_	0.84, s	
26	16.3, CH_3_	0.85, s	
27	14.3, CH_3_	0.94, s	
28	16.9, CH_3_	0.87, s	
29a	109.5, CH_2_	4.69, s	
29b		4.57, s	
30	18.1, CH_3_	1.68, s	
1′	173.0, C		
2′	36.7, CH_2_	2.45, t (7)	
3′	25.2, CH_2_	1.62, m	
4′	29.5, CH_2_	1.18–1.28 ^a^, m	
5′	29.6, CH_2_	1.18–1.28 ^a^, m	
6′	29.7, CH_2_	1.18–1.28 ^a^, m	
7′	29.9, CH_2_	1.18–1.28 ^a^, m	
8′	29.8, CH_2_	1.18–1.28 ^a^ m	
9′	29.8, CH_2_	1.18–1.28 ^a^, m	
10′	29.8, CH_2_	1.18–1.28 ^a^, m	
11′	29.7, CH_2_	1.18–1.28 ^a^, m	
12′	32.1, CH_2_	1.32 ^a^, m	
13′	22.8, CH_2_	0.79	
14′	14.7, CH_3_	0.88, t (7.0)	
	OH	2.94, br	

^a^ Partially overlapped signal.

**Table 3:** ^1^H NMR (400 MHz) and ^13^C NMR (100 MHz) Data for Compound **3** in CDCl_3._

Position	δ_C_ (ppm)	δ_H_ (ppm) (*J* in Hz)	HMBC (^1^H → ^13^C)
1	130.7, C		
2	114.7, CH	6.69, (br s)	
3	144.7, C		
4	147.0, C		
5	113.1, CH	6.63, d (7.9)	
6	123.6, CH	6.84, d (7.9)	
7	76.9, C		
8a	42.4, CH_2_	3.10, d (13.7)	1, 2, 6, 7, 8′, 9′
8b		2.91, d (13.7)	1, 2, 6, 7, 8′, 9′
9	179.1, C		
1′	126.5, C		
2′	111.9, CH	6.60, (br s)	
3′	147.0, C		
4′	145.4, C		
5′	114.9, CH	6.82, d (7.9)	
6′	121.9, CH	6.62, d (7.9)	
7′ a	31.9, CH_2_	2.59, dd (9.4, 4.6)	1′, 2′, 6′, 8′, 9′, 7, 8
7′ b		2.49, dd (9.1, 4.5)	1′, 2′, 6′, 8′, 9′, 7, 8
8′	44.2, CH	2.52, m	1, 7, 8, 9, 2′, 6′, 7′, 9′
9′ a	70.7, CH_2_	4.04, dd (8.9, 6.7)	7, 9, 7′, 8′
9′ b		3.99, dd (8.9, 7.8)	7, 9, 7′, 8′
4-OCH_3_	56.4, CH_3_	3.85, s	3, 4
4′-OCH_3_	56.3, CH_3_	3.64, s	3′, 4′
	OH	5.5–5.8, (broad)	

## Data Availability

Not applicable.

## Supplementary Materials

The following supporting information can be downloaded at: https://www.mdpi.com/article/10.3390/molecules28166077/s1.

## Sample Availability

Samples of the compound Maytendhofarene is available from the corresponding authors.
