# Supplementary material for: Isolation and Identification of Phytocompounds from Maytenus dhofarensis and Their Biological Potentials
Source: Molecules. 2023 Aug 15;28(16):6077. doi: 10.3390/molecules28166077 (PMC10459021; doi:10.3390/molecules28166077)
Supplement: Supplementary file 1 [file molecules-28-06077-s001.zip › molecules-2536190-supplementary.pdf]

Isolation and Identification of Phytocompounds from *Maytenus dhofarensis* and Their Biological Potentials

Fatma Al-Rubaiai[1], Zakiya Zahran Al-Shariqi[1], , Khalsa S. Al-Shabibi[1], John Husband[1], Asmaa M. Al-Hattali[1], Marcia Goettert[2], Stefan Laufer[2,3], Younis Baqi[1], and Syed Imran Hassan[1*] Majekodunmi O. Fatope[1*]

[1] Department of Chemistry, College of Science, Sultan Qaboos University, PO Box 36, Al Khod, Muscat 123, Oman; F.A-R: s87268@student.squ.edu.om; Z.Z.A-S.: zaak338@hotmail.com; K.S.A-S: pulslife93@gmail.com; J.H.: johnh@squ.edu.om; A.M.A-H: asmaa.alhattali@live.com; Y.B.: baqi@squ.edu.om; S.I.H.: s.hasan@squ.edu.om; M.O.F: majekmof@gmail.com

[2]Department of Pharmaceutical and Medicinal Chemistry, Institute of Pharmacy, Eberhard Karls Universität Tübingen, D-72076 Tübingen, Germany; M.G: marcia.goettert@univates.br; S.L: stefan.laufer@uni-tuebingen.de

[3]Tübingen Center for Academic Drug Discovery (TüCAD2), D-72076 Tübingen, Germany; stefan.laufer@uni-tuebingen.de

Supporting Information

*Correspondence: S.I.H.: s.hasan@squ.edu.om; M.O.F: majekmof@gmail.com

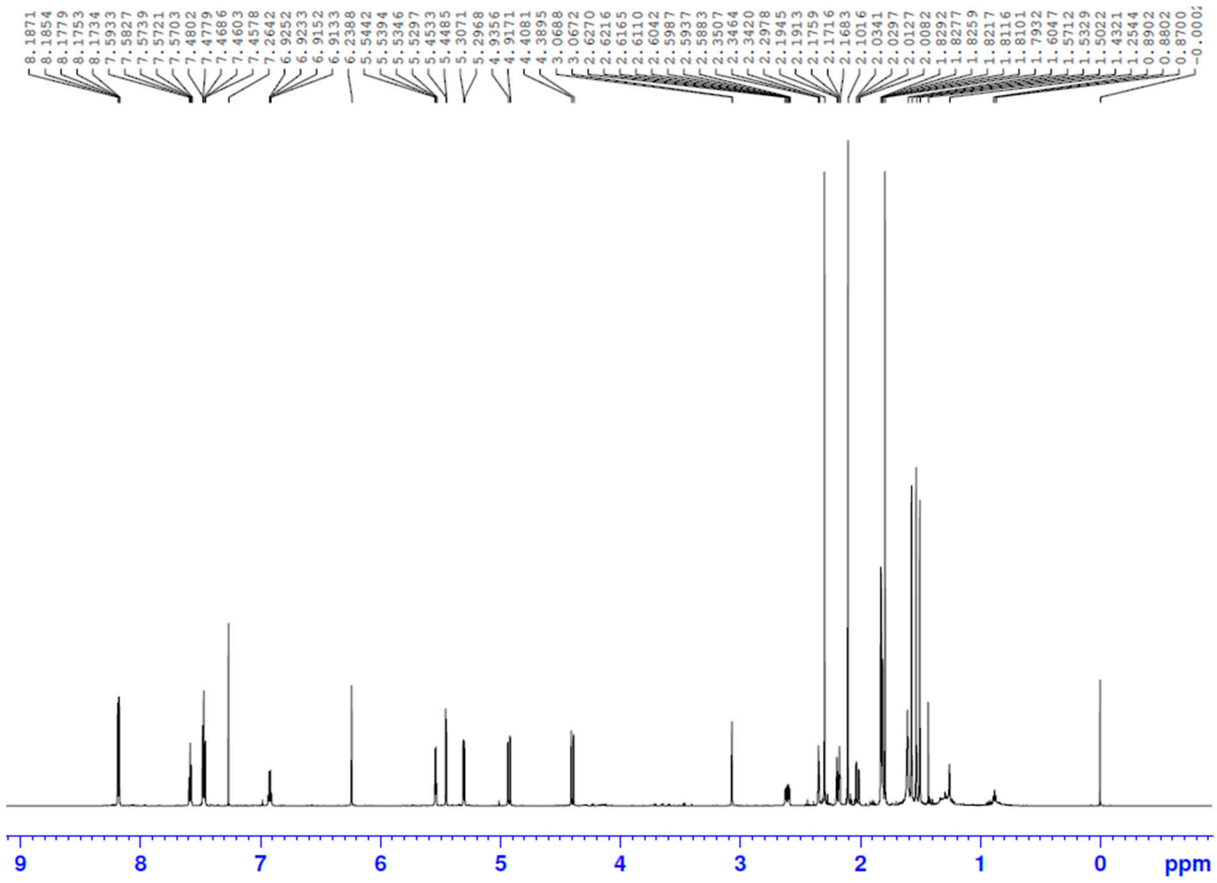

Figure S1: ^{1}H NMR spectrum (700 MHz, Chloroform-*d*) of compound **1**

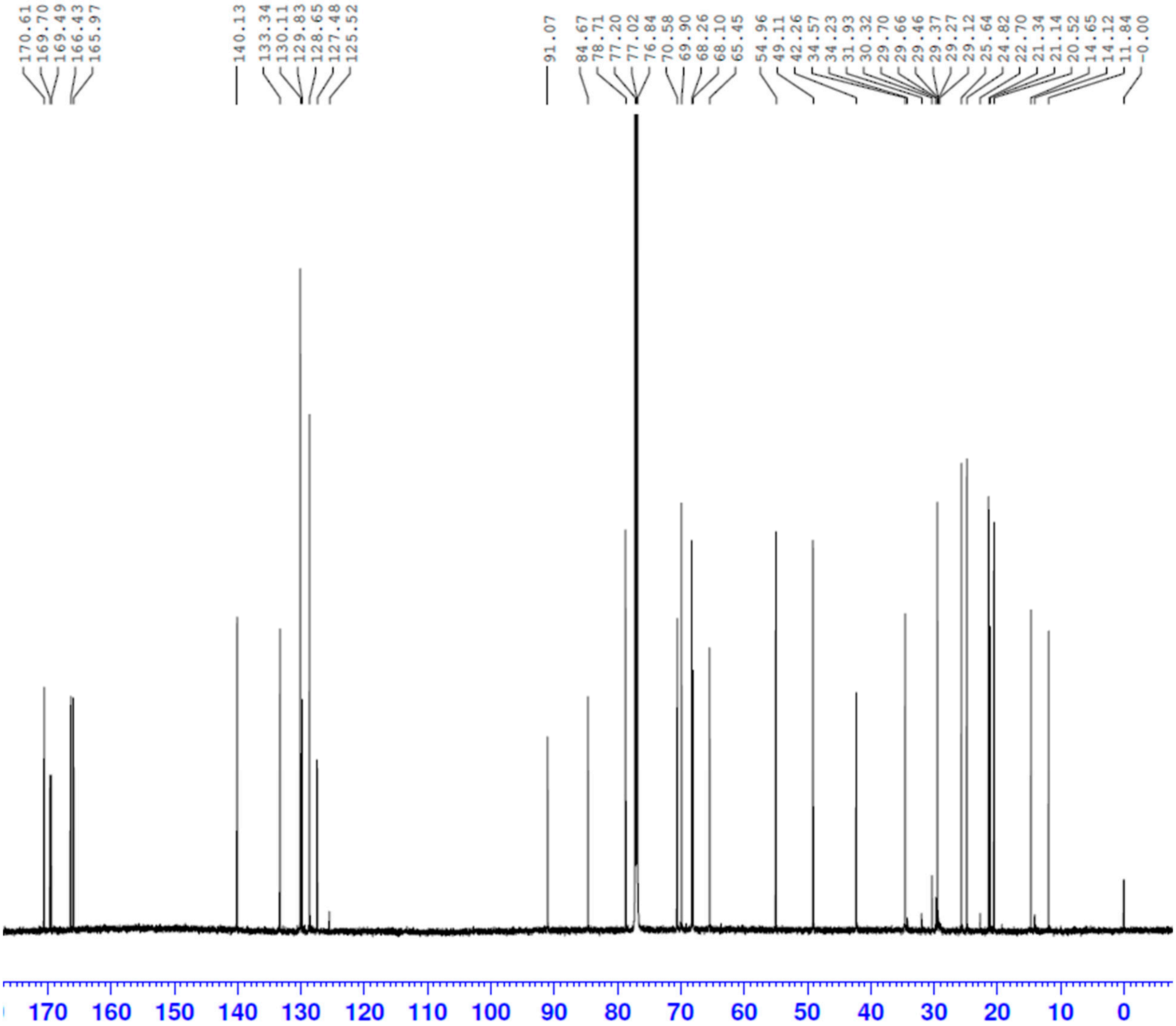

Figure S2: ^{13}C NMR spectrum (176 MHz, Chloroform-*d*) of compound **1**

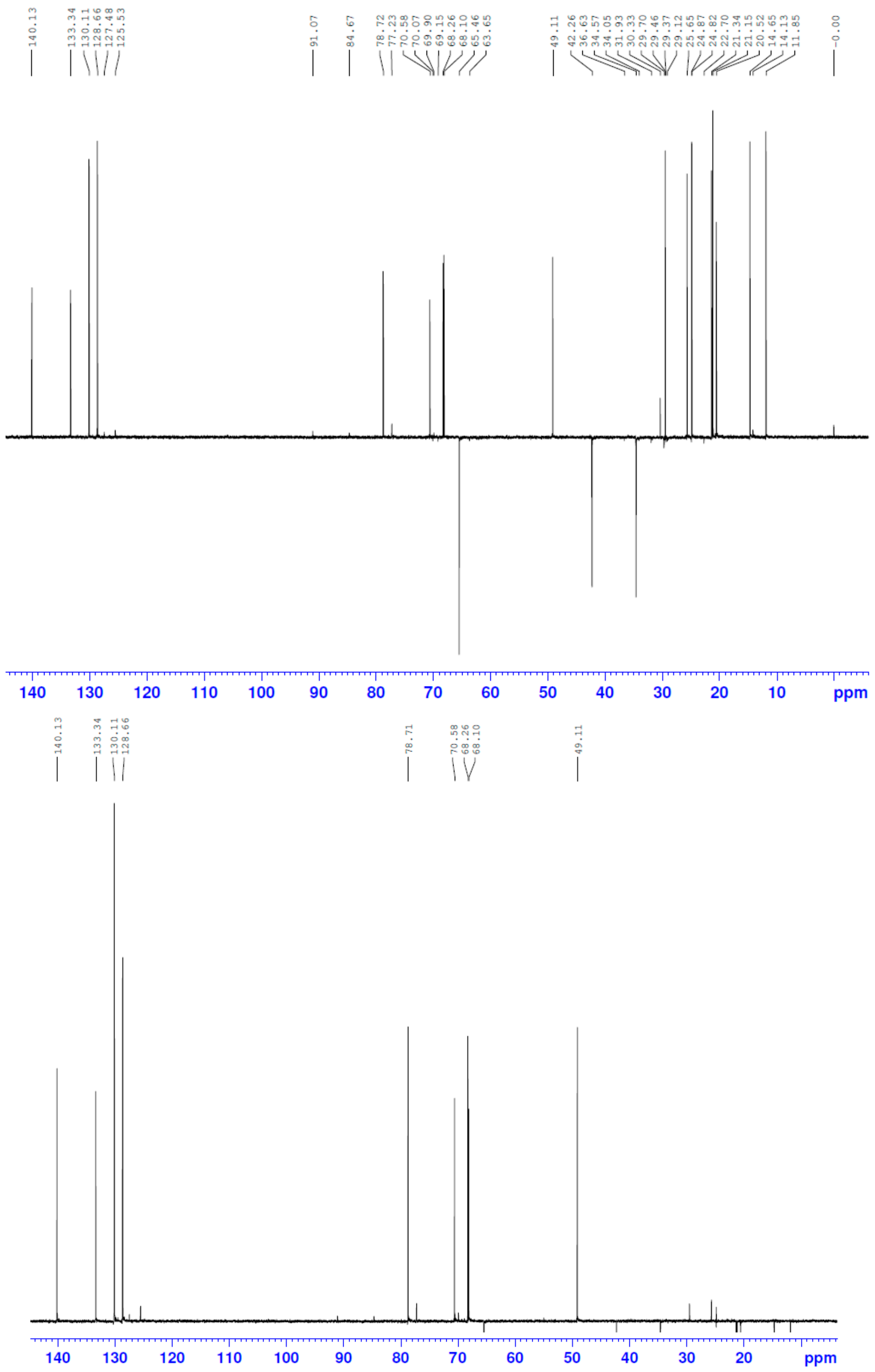

Figure S3: DEPT-135 and 90 spectra (176 MHz, Chloroform-*d*) of compound **1**

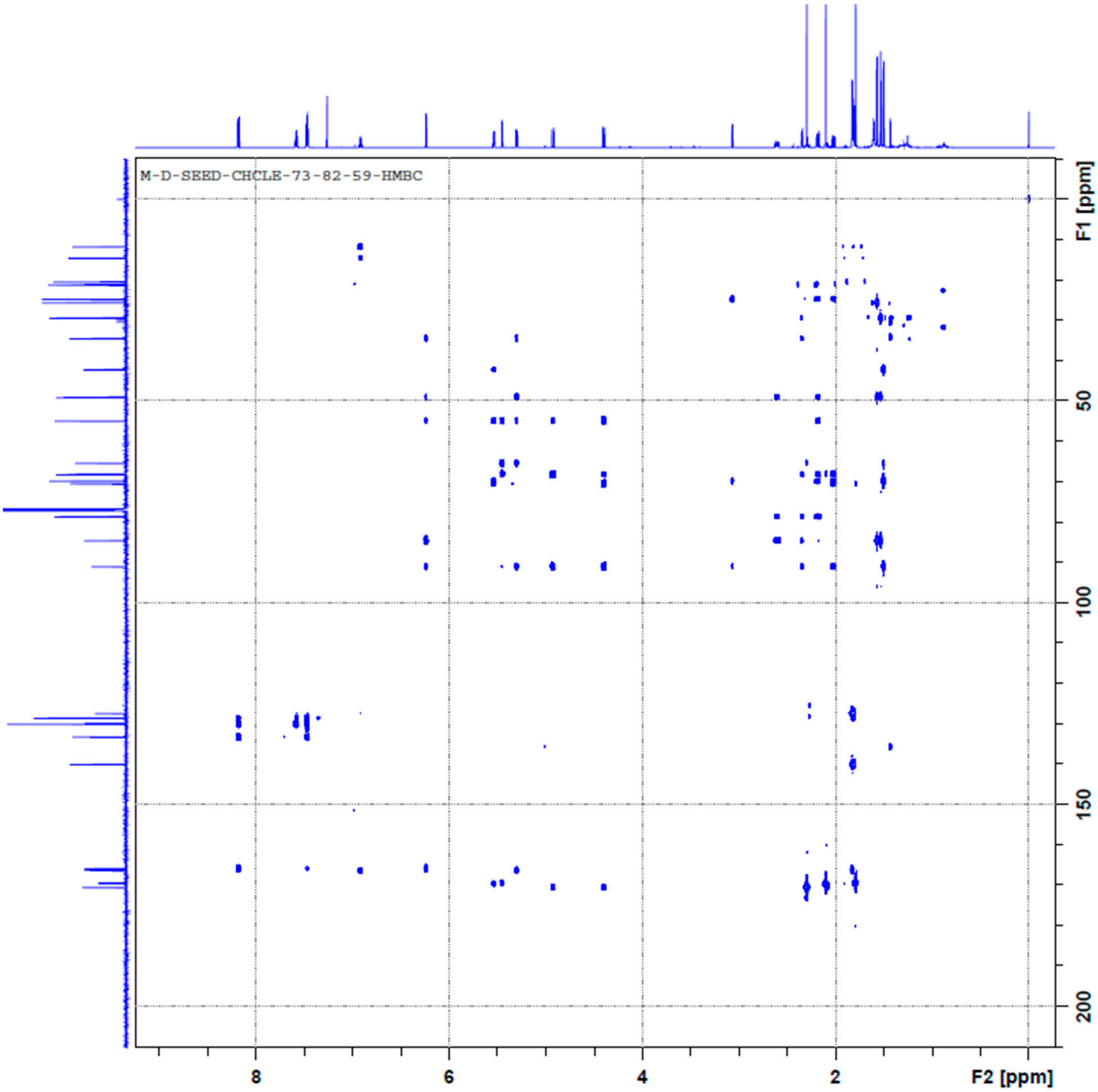

Figure S4: HMBC spectrum of compound **1**

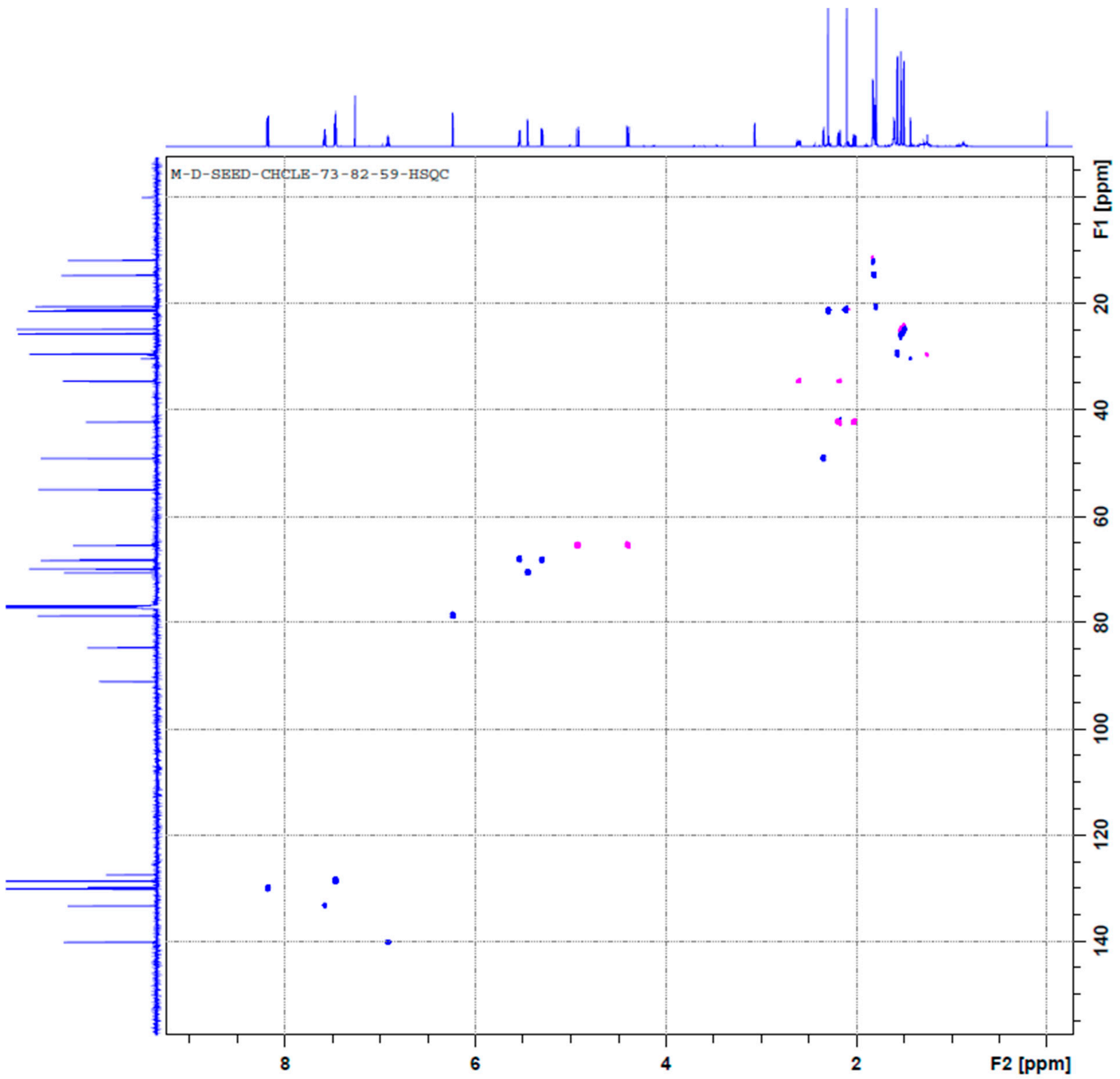

Figure S5: HMBC spectrum of compound **1**

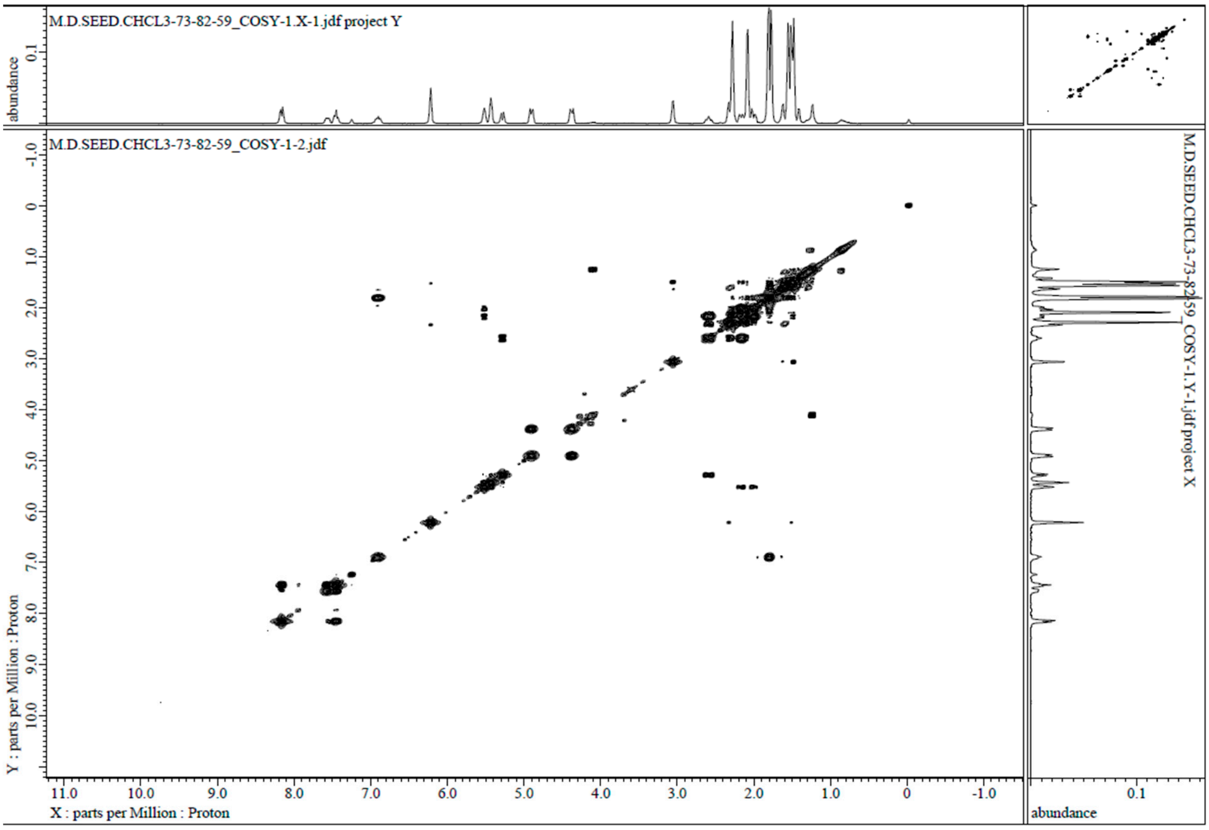

Figure S6: ^{1}H-^{1}H COSY spectrum of compound **1**

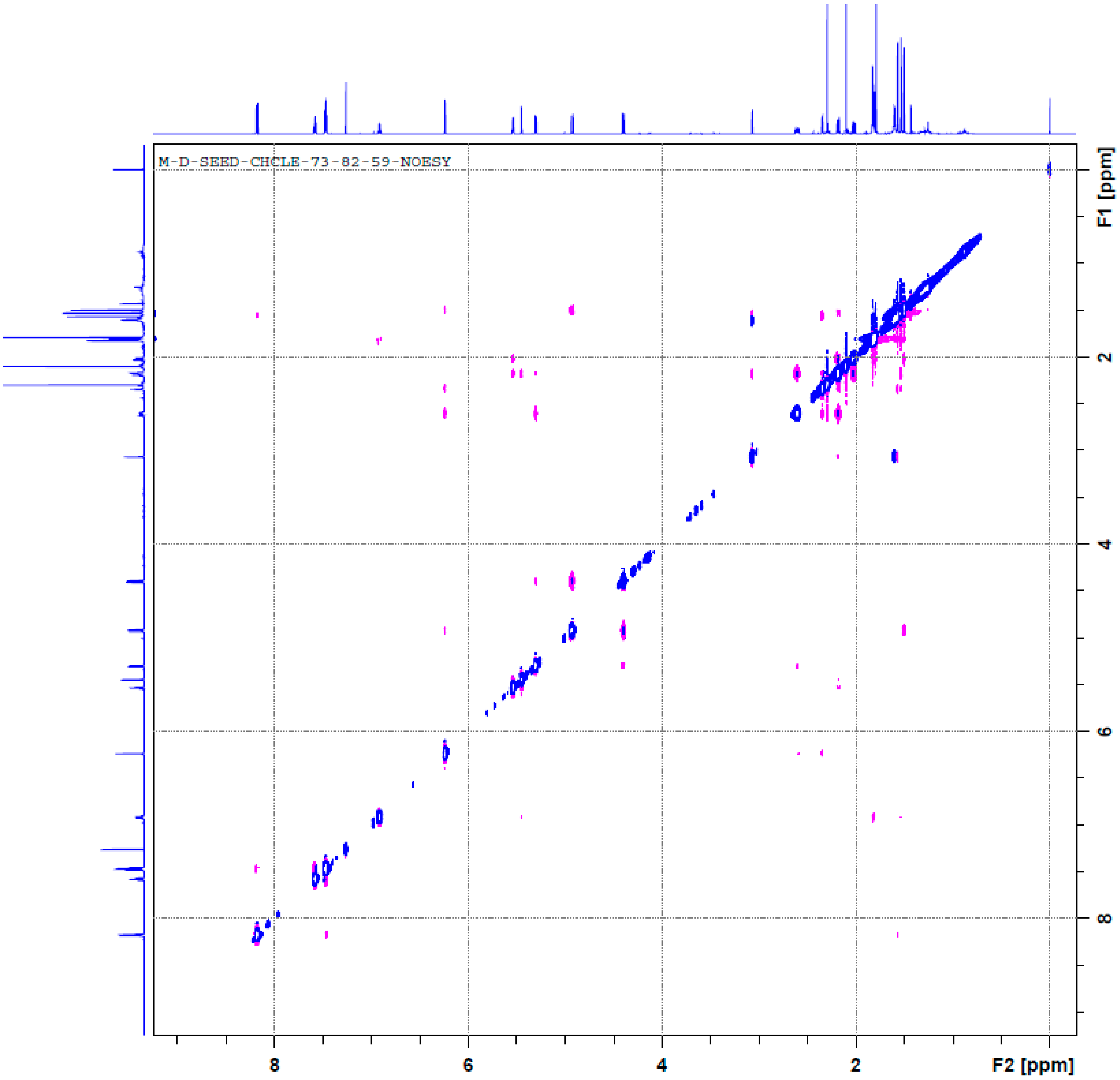

Figure S7: NOESY spectrum of compound **1**

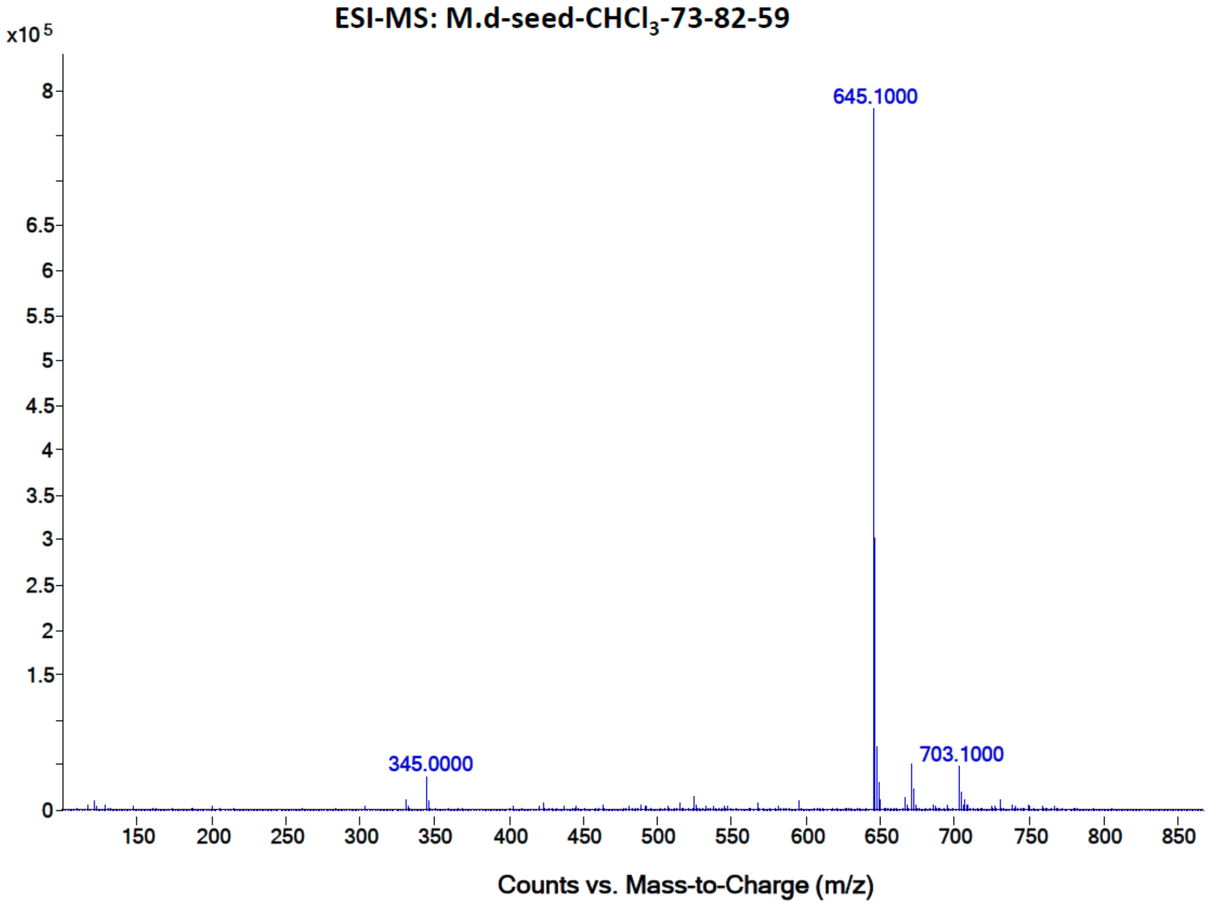

Figure S8: ESI MS spectrum of compound **1**

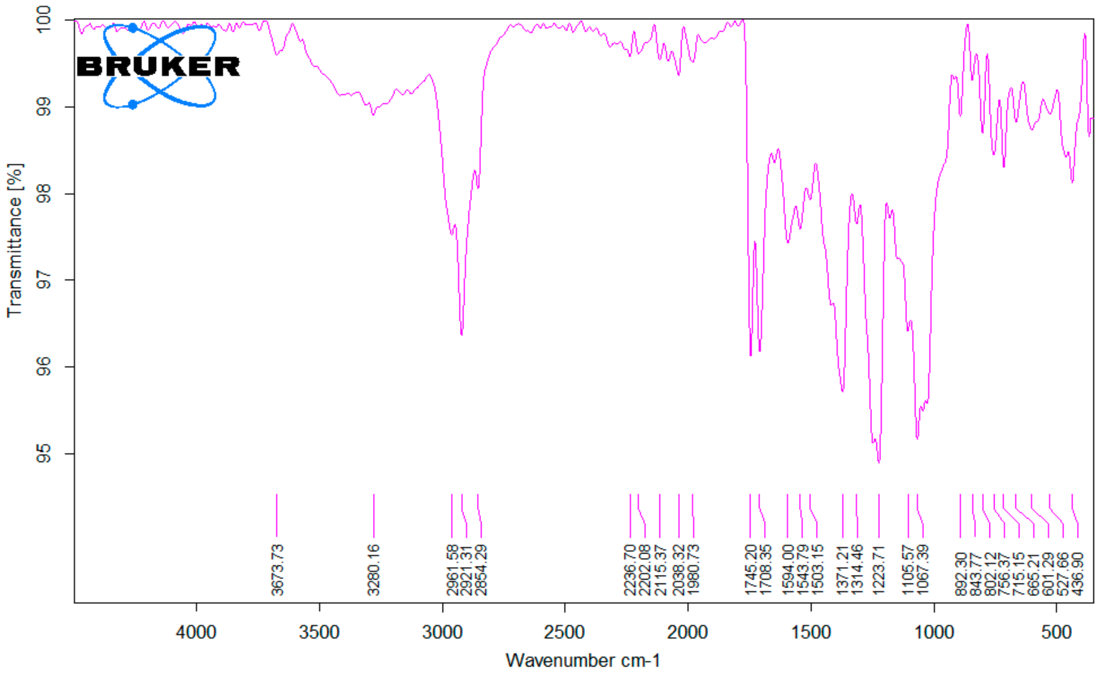

C:\Data\2023\MARCH 2023\I-325\M-D-SEED-CHCl3-73-82-59-1.0	M-D-SEED-CHCl3-73-82-59-1	30-Mar-23

Page 1 of 1

Figure S9: FT IR spectrum of compound **1**

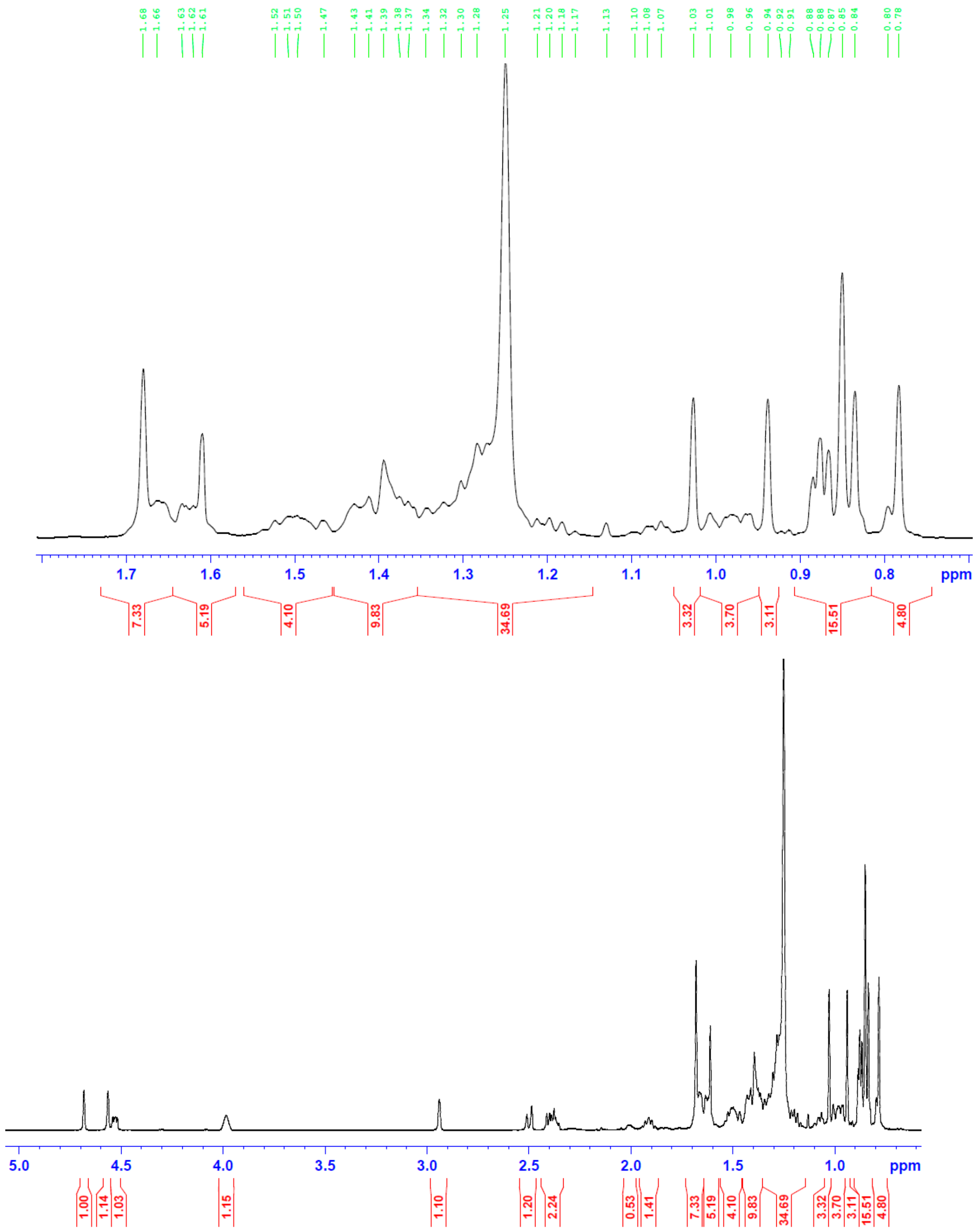

Figure S10: ^{1}H NMR (700 MHz, Chloroform-*d*) of compound **2**

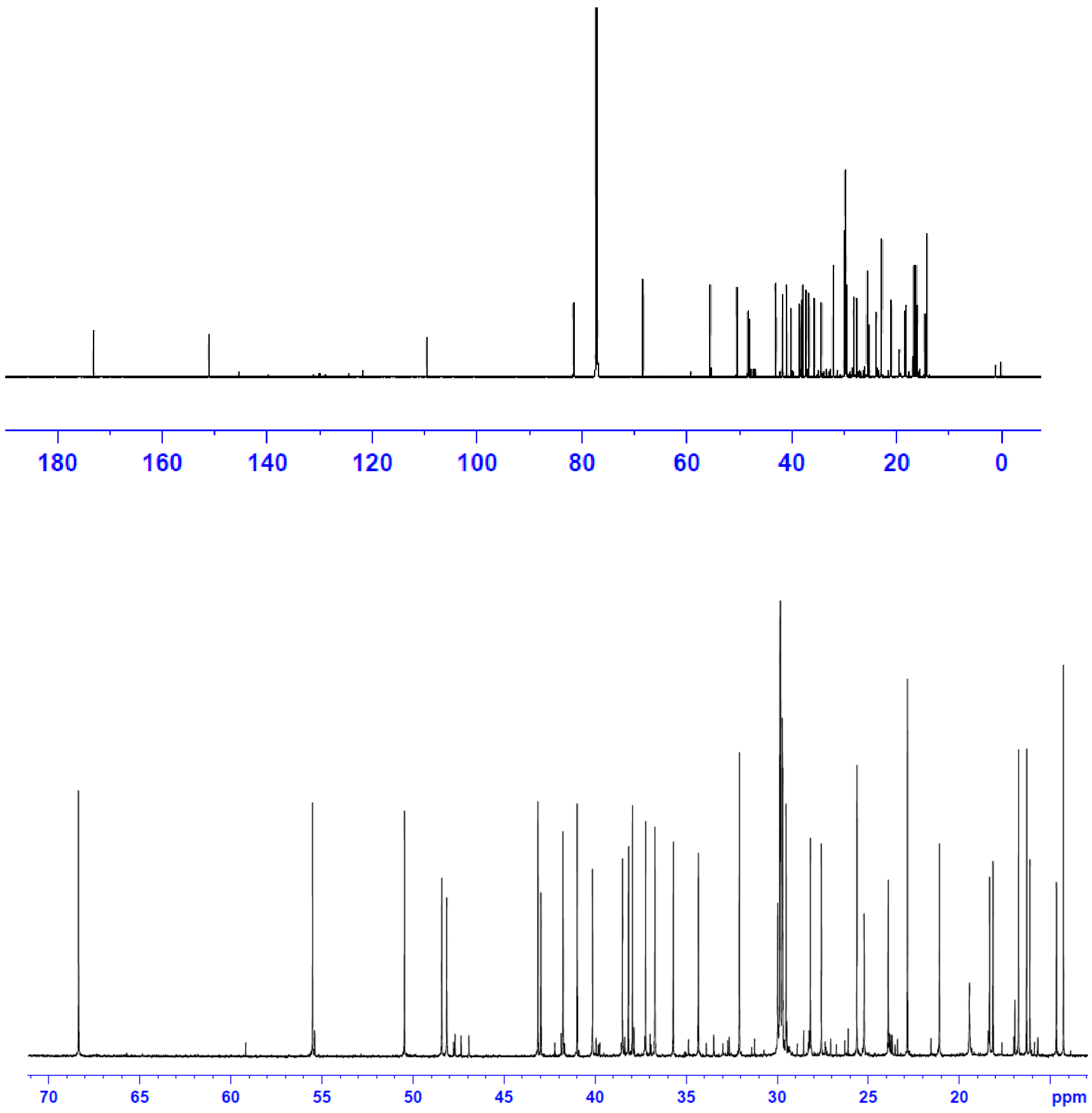

Figure S11: ^{13}C NMR (176 MHz, Chloroform-*d*) of compound **2**

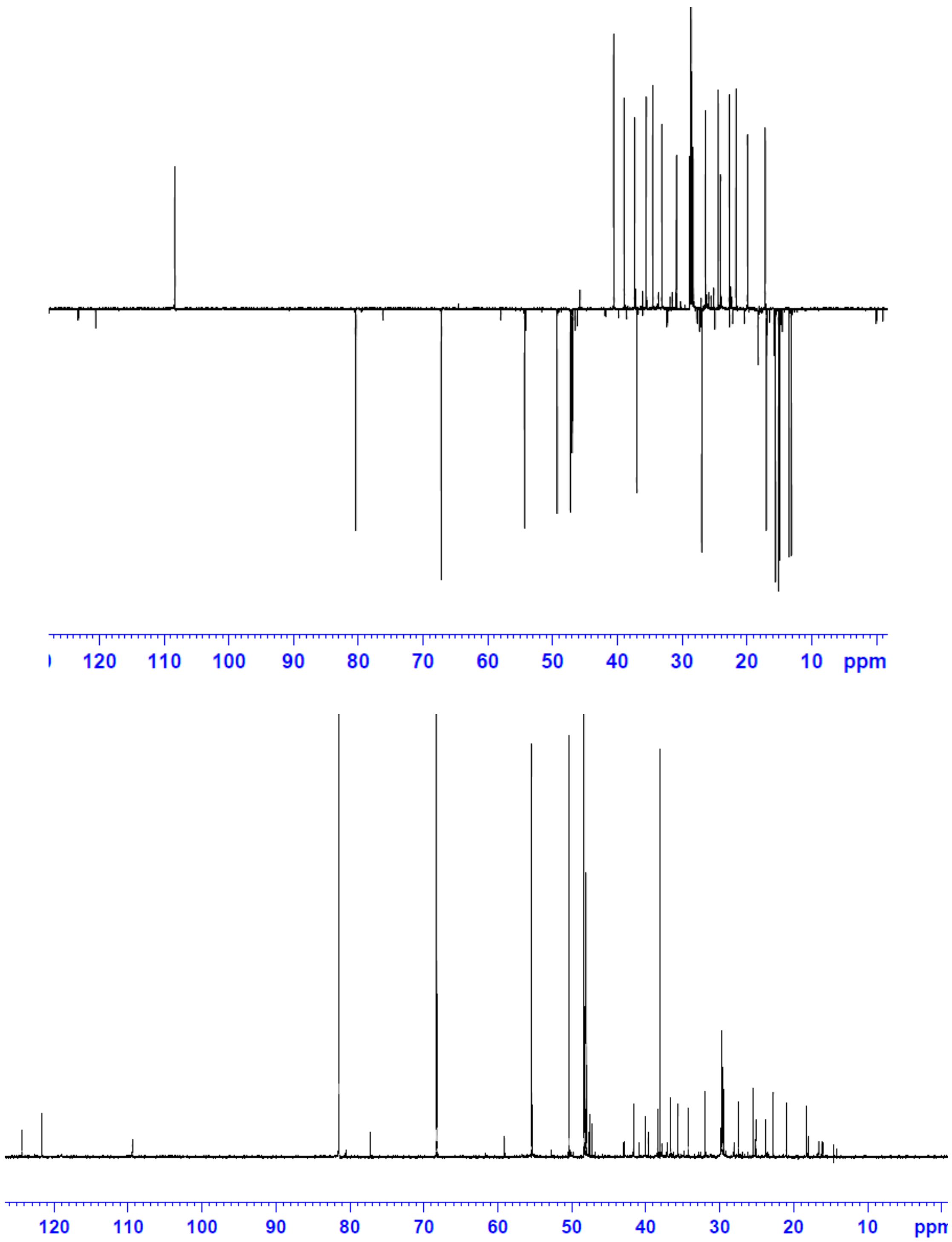

Figure S12: DEPT-135 and 90 (176 MHz, Chloroform-*d*) compound **2**

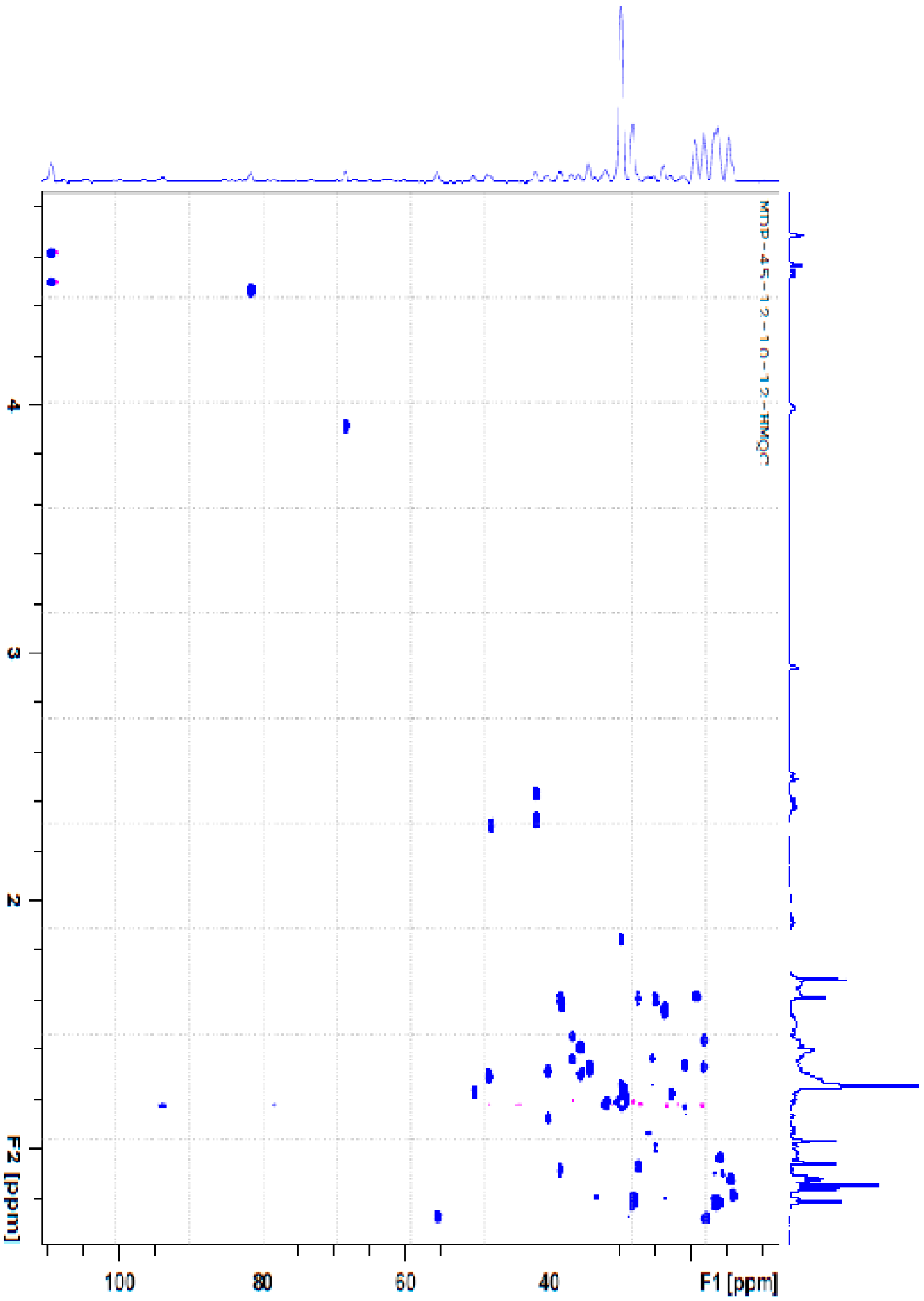

Figure S13: HMQC of compound **2**

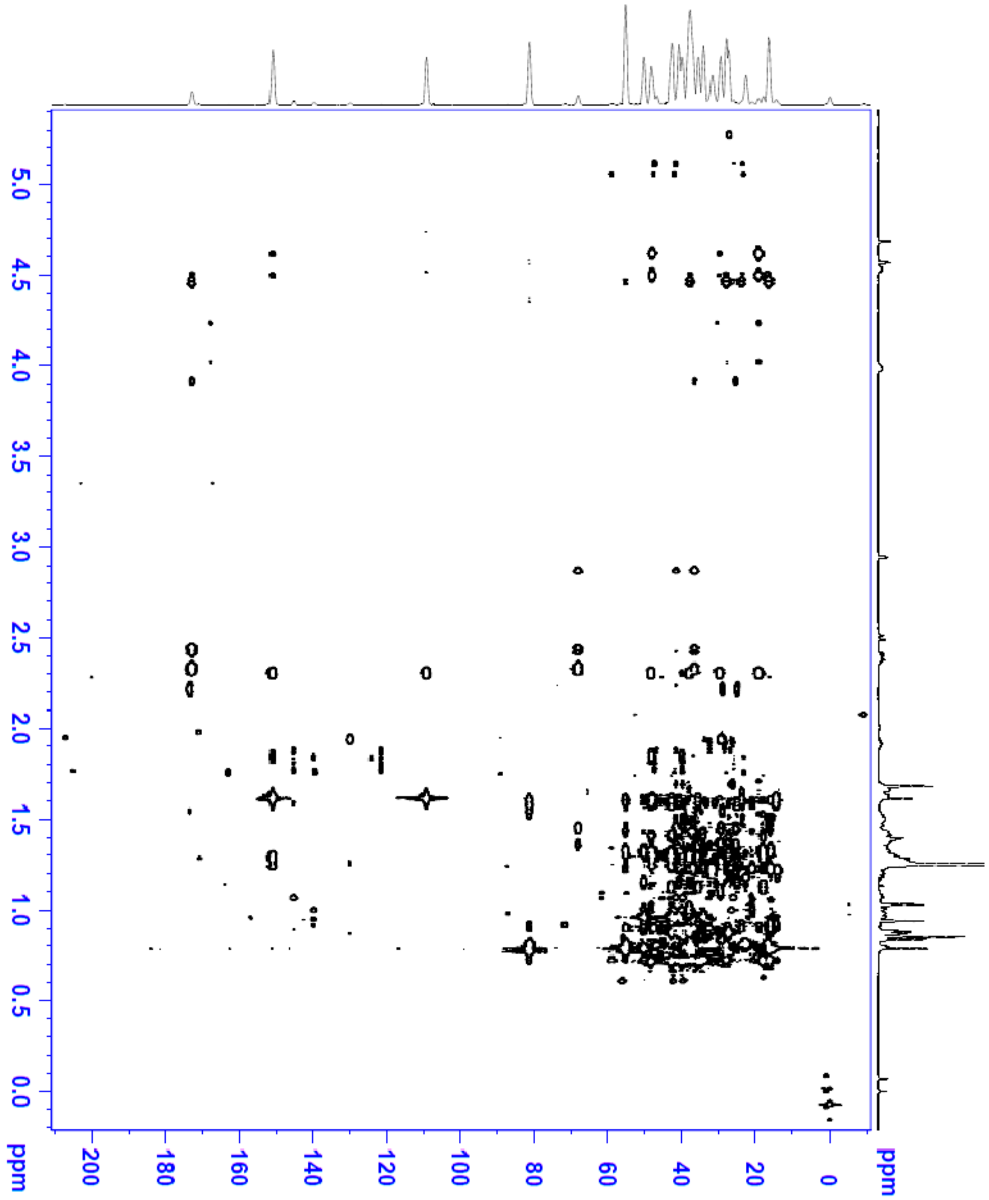

Figure S14: HMBC of compound **2**

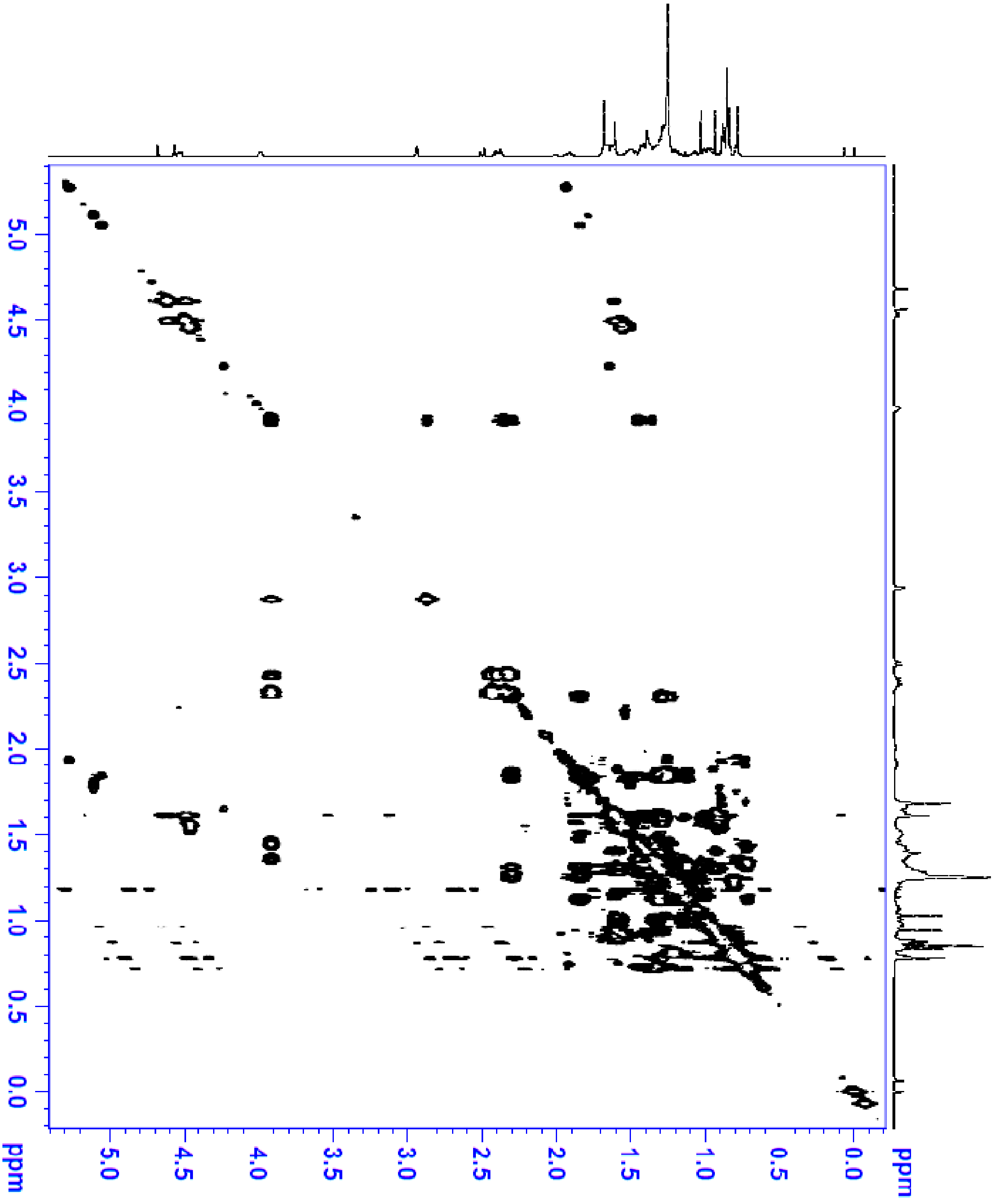

Figure S15: ^{1}H-^{1}H COSY compound **2**

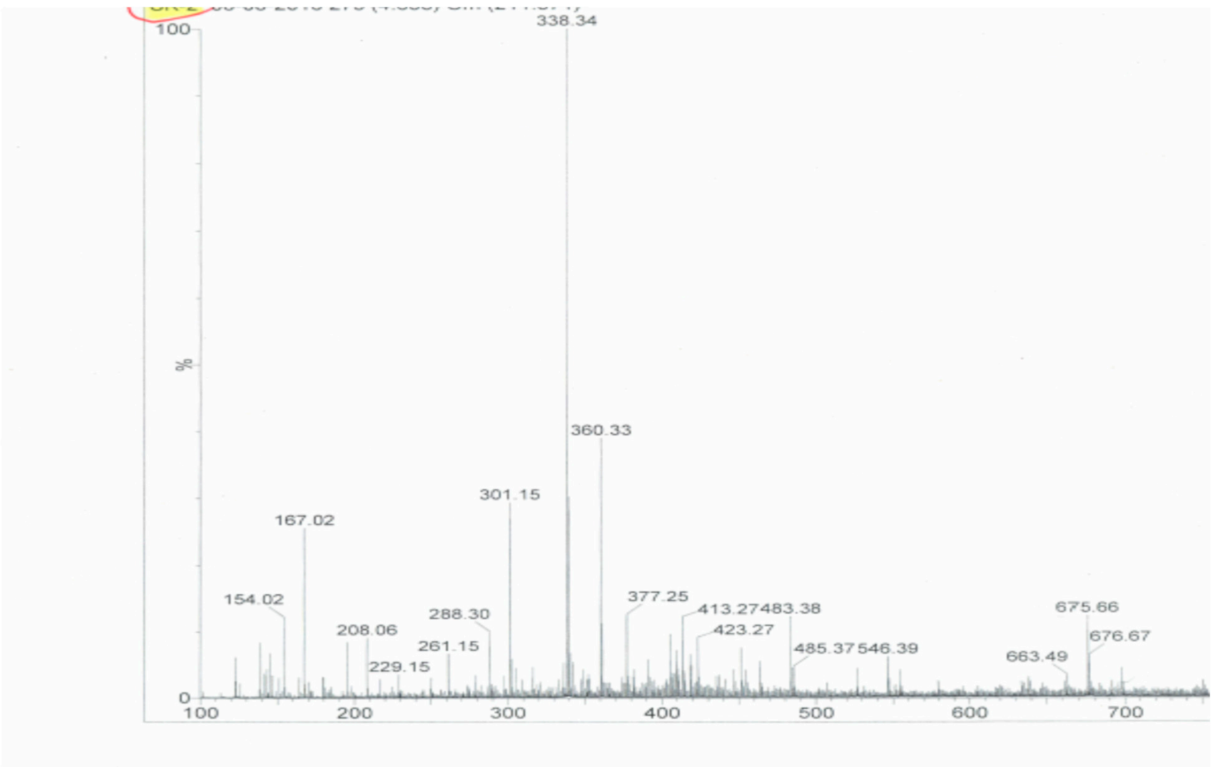

Figure S16: ESI MS spectrum of compound **2**

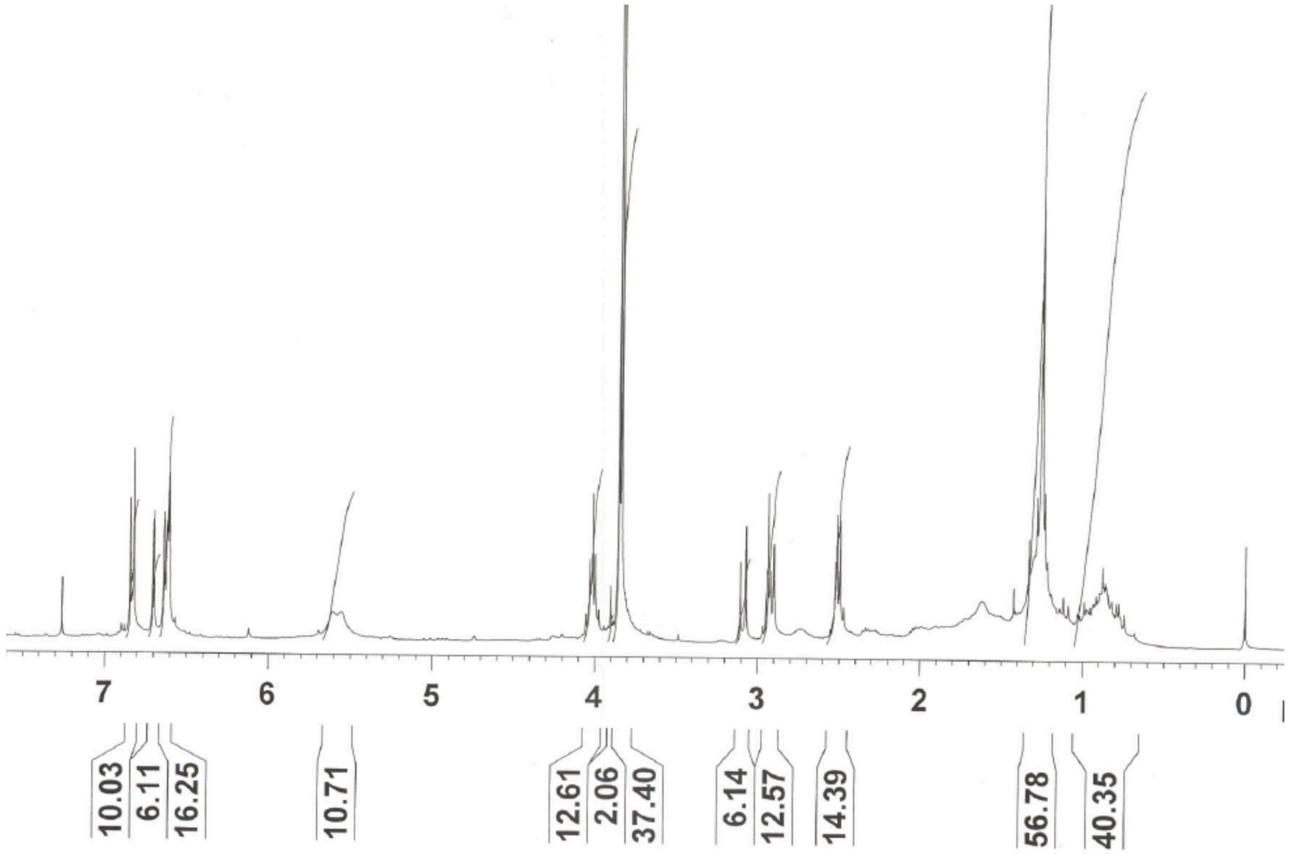

Figure S17: ^{1}H NMR (400 MHz, Chloroform-*d*) of compound **3**

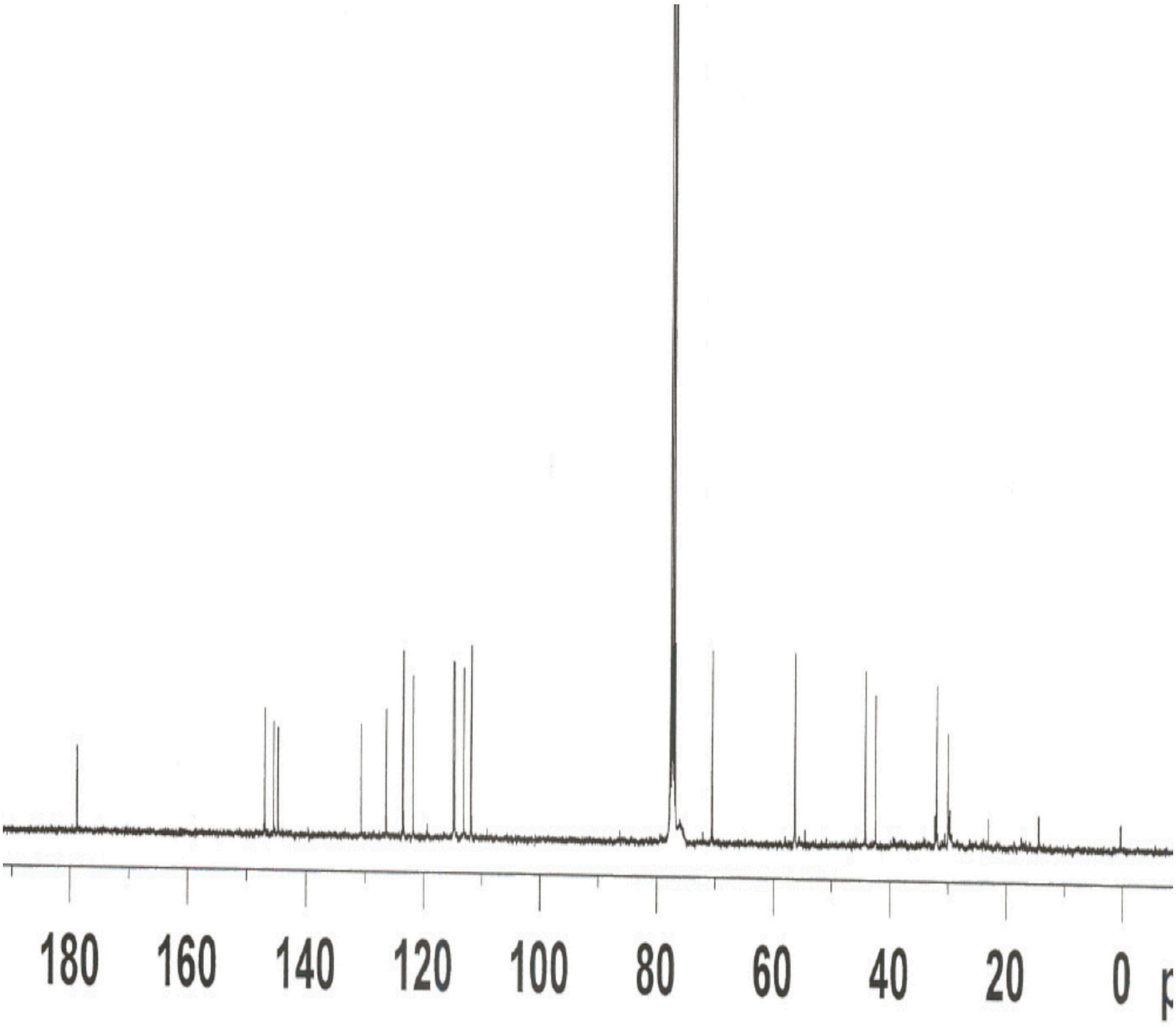

Figure S18: ^{13}C NMR (100 MHz, Chloroform-*d*) of compound **3**

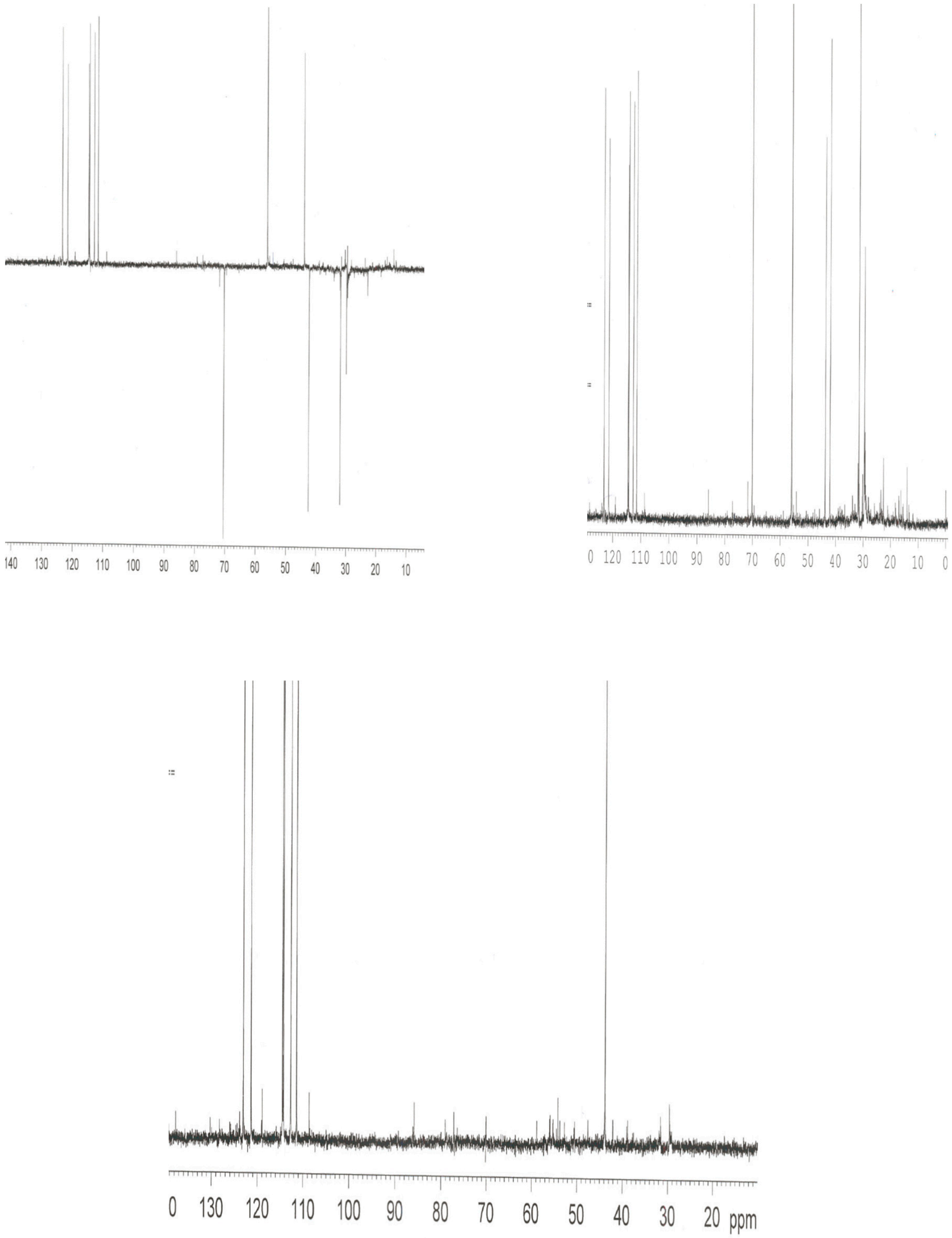

Figure S19: DEPT-135, 90 and 45 (100 MHz, Chloroform-*d*) of compound **3**

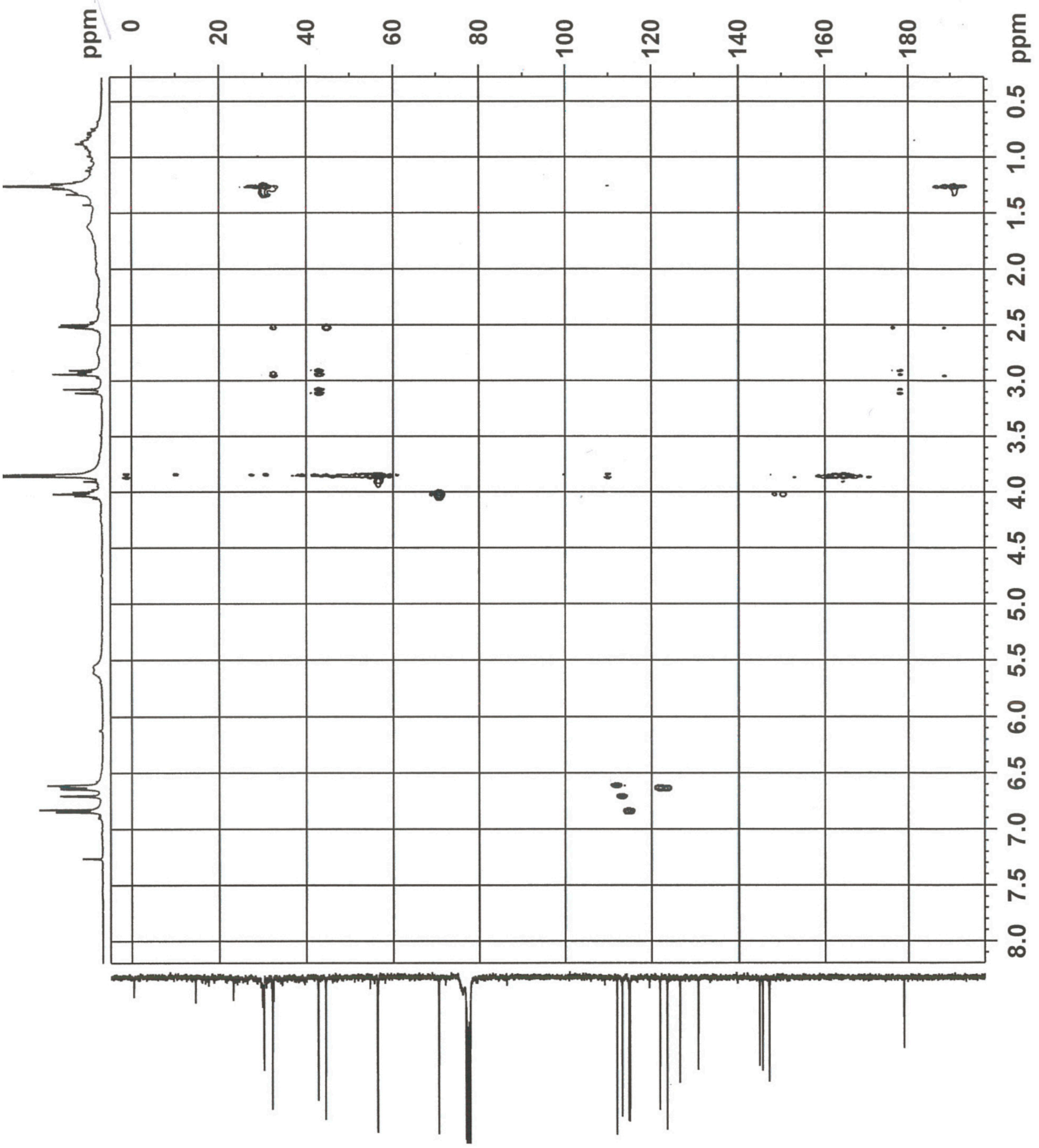

Figure S20: HMQC of compound **3**

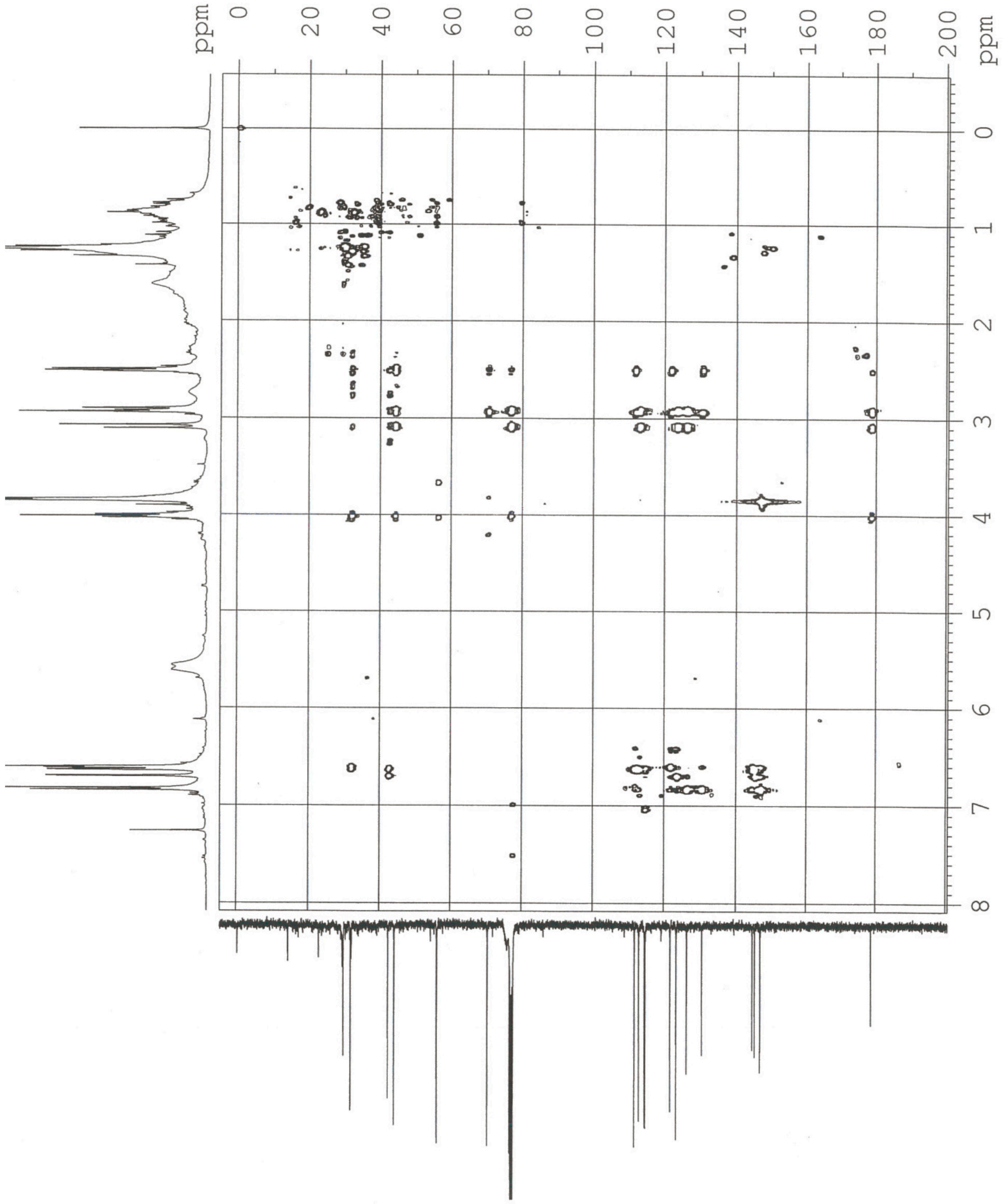

Figure S21: HMBC of compound **3**

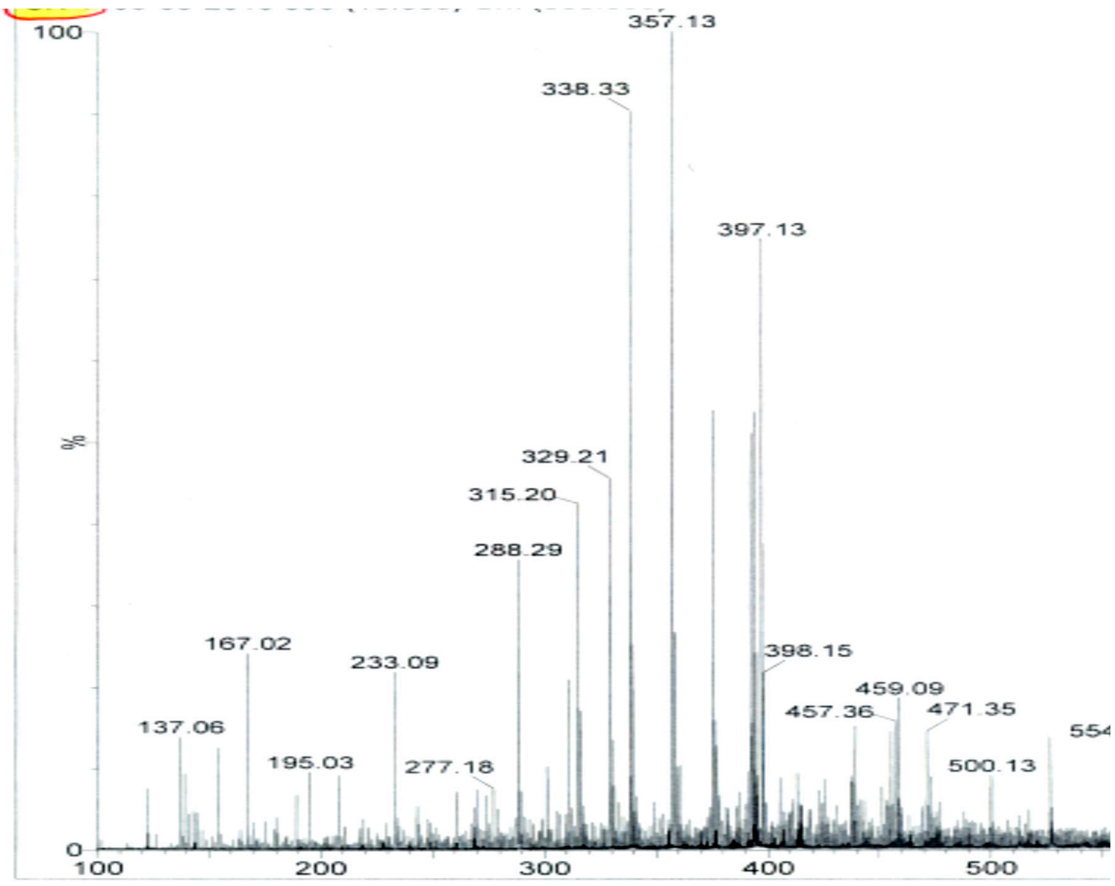

Figure S22: ESI MS spectrum of compound **3**

Table S1: Comparison of the predicted chemical shifts for diastereoisomers 9*R* and 9*S* of compound 3. Atom numbering is as in the Manuscript.

Position	9*R*		9*S*	
	δ_C[a]	δ_H[a]	δ_C[a]	δ_H[a]
1	71.7	4.51	70.9	4.21
2	78.8	5.25	73.0	5.32
3	37.6	2.37, 2.03	38.1	2.24, 2.23
4	74.2		73.7	
5	89.3		88.9	
6	78.1	6.12	80.9	6.54
7	47.5	2.27	46.8	2.45
8	26.8	2.83, 1.86	32.6	2.59, 2.31
9	67.5	5.67	76.3	4.40
10	58.9		58.5	
11	82.7		83.5	
12	27.2	1.47	26.7	1.12
13	24.2	1.37	22.0	1.49
14	27.3	1.48	24.4	1.64
15	65.9	4.58, 4.40	64.6	4.30, 3.98
2`	149.5		149.6	
3`	133.3		133.0	
4`	135.2	7.58	135.1	7.74
5`	121.2	7.13	120.3	7.30
6`	143.7	8.39	143.3	8.60
7`	133.3	7.29	128.5	7.26
8`	133.2		135.7	
9`	171.6		172.0	
10`	16.1	2.36	15.8	2.60
11`	20.0	2.49	18.7	2.48
OAc-6	21.1	2.19	20.8	2.29
	167.8		168.7	
OAc-9	21.2	2.17	18.3	2.40
	168.3		168.43	
OAc-15	21.4	2.16	20.6	2.24
	168.9		169.6	

[a]Calculated from the magnetic shielding constants (σ) computed using the gauge including atomic orbitals (GIAO) method:

$$\delta = \frac{b - \sigma}{-m}$$

For the values shown in the table *m* and *b* have been optimized by a simple linear regression to best fit the measured values. Optimized values are:

9*R*: b_C = 187.224; m_C = -1.0705 and b_H = 31.9556; m_H = -1.0952

9*S*: b_C = 186.032; m_C = -1.0616 and b_H = 31.9831; m_H = -1.0745

Table S2: Results of the DP4+ analysis of the comparison of the 9*R* and 9*S* predicted shielding tensors to the measured chemical shifts for compound **3**. Diastereoisomers 9*R* is clearly favoured.

Data set	9*R*	9*S*
sDP4+ (H data)	100.00%	0.00%
sDP4+ (C data)	99.43%	0.57%
sDP4+ (all data)	100.00%	0.00%
uDP4+ (H data)	97.33%	2.67%
uDP4+ (C data)	99.99%	0.01%
uDP4+ (all data)	100.00%	0.00%
DP4+ (H data)	100.00%	0.00%
DP4+ (C data)	100.00%	0.00%
DP4+ (all data)	100.00%	0.00%

Table S3: Predicted chemical shifts for diastereoisomers of compound **3**.

Position	δ$_C$			δ$_H$		
	Exp	Calc 7*R*-8'*R*	Calc 7*S*-8'*R*	Exp	Calc 7*R*-8'*R*	Calc 7*S*-8'*R*
1	130.7	136.8	140.0			
2	114.7	112.9	110.1	6.69	6.55	7.22
3	144.7	146.1	147.3			
4	147.0	146.7	145.4			
5	113.1	106.6	107.8	6.63	6.76	6.71
6	123.6	119.1	114.6	6.84	7.24	6.66
7	76.9	78.3	78.5			
8	42.4	49.9	48.5	3.10	2.80	3.04
				2.91	2.74	2.21
9	179.1	170.6	170.6			
1'	126.5	134.7	133.8			
2'	111.9	113.0	111.4	6.6	6.88	6.53
3'	147.4	147.5	148.0			
4'	145.4	145.3	145.1			
5'	114.9	106.5	107.0	6.82	6.44	6.57
6'	121.9	119.5	119.3	6.62	6.21	6.43
7'	31.9	33.3	34.4	2.59	3.41	2.25
				2.49	1.45	2.20
8'	44.20	44.2	49.5	2.52	2.41	2.24
9'	70.7	71.1	72.5	3.99	3.70	3.94
				4.04	3.85	4.54

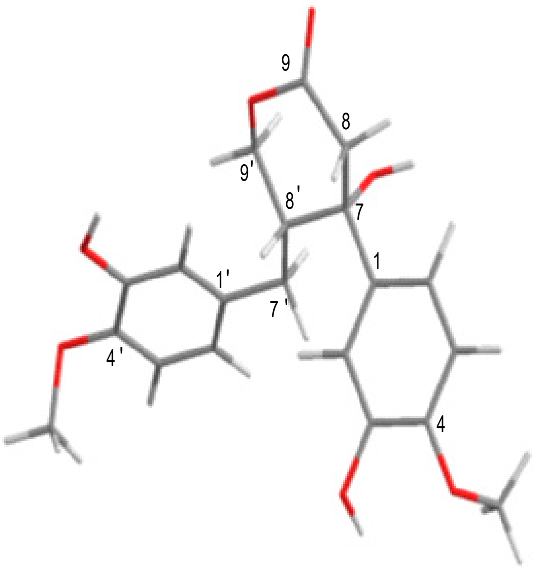

Figure S23. Spatial model of arrangement of groups around stereogenic C-8’ and C-7 in compound **3**
